# Short- and long-term T cell and antibody responses following dexamethasone treatment in COVID-19

**DOI:** 10.1172/jci.insight.166711

**Published:** 2023-04-24

**Authors:** Charlotte Thibeault, Lara Bardtke, Kanika Vanshylla, Veronica di Cristanziano, Kirsten A. Eberhardt, Paula Stubbemann, David Hillus, Pinkus Tober-Lau, Parnika Mukherjee, Friederike Münn, Lena J. Lippert, Elisa T. Helbig, Tilman Lingscheid, Fridolin Steinbeis, Mirja Mittermaier, Martin Witzenrath, Thomas Zoller, Pa-COVID study group, Florian Klein, Leif E. Sander, Florian Kurth

**Affiliations:** 1 Department of Infectious Diseases, Respiratory and Critical Care Medicine, Charité-Universitätsmedizin Berlin, corporate member of Freie Universität Berlin and Humboldt-Universität zu Berlin, Berlin, Germany.; 2 Institute of Virology, Faculty of Medicine and University Hospital Cologne, University of Cologne, Cologne, Germany.; 3 German Center for Infection Research, Partner Site Bonn-Cologne, Cologne, Germany.; 4 Division of Hygiene and Infectious Diseases, Institute of Hygiene and Environment, Hamburg, Germany.; 5 Department of Tropical Medicine, Bernhard Nocht Institute for Tropical Medicine and Department of Medicine I, University Medical Center Hamburg-Eppendorf, Hamburg, Germany.; 6 Berlin Institute of Health at Charité, Berlin, Germany.; 7 German Center for Lung Research (DZL), Berlin, Germany.; 8 The Pa-COVID study group is detailed in Supplemental Acknowledgments.; 9 Center for Molecular Medicine Cologne (CMMC), University of Cologne, Cologne, Germany.

**Keywords:** Immunology, Infectious disease, Adaptive immunity, Immunotherapy, T cells

## Abstract

**BACKGROUND:**

After its introduction as standard-of-care for severe COVID-19, dexamethasone has been administered to a large number of patients globally. Detailed knowledge of its impact on the cellular and humoral immune response to SARS-CoV-2 remains scarce.

**METHODS:**

We included immunocompetent individuals with (a) mild COVID-19, (b) severe COVID-19 before introduction of dexamethasone treatment, and (c) severe COVID-19 infection treated with dexamethasone from prospective observational cohort studies at Charité-Universitätsmedizin Berlin, Germany. We analyzed SARS-CoV-2 spike–reactive T cells, spike-specific IgG titers, and serum neutralizing activity against B.1.1.7 and B.1.617.2 in samples ranging from 2 weeks to 6 months after infection. We also analyzed BA.2 neutralization in sera after booster immunization.

**RESULTS:**

Patients with severe COVID-19 and dexamethasone treatment had lower T cell and antibody responses to SARS-CoV-2 compared with patients without dexamethasone treatment in the early phase of disease, which converged in both groups before 6 months after infection and also after immunization. Patients with mild COVID-19 had comparatively lower T cell and antibody responses than patients with severe disease, including a lower response to booster immunization during convalescence.

**CONCLUSION:**

Dexamethasone treatment was associated with a short-term reduction in T cell and antibody responses in severe COVID-19 when compared with the nontreated group, but this difference evened out 6 months after infection. We confirm higher cellular and humoral immune responses in patients after severe versus mild COVID-19 and the concept of improved hybrid immunity upon immunization.

**FUNDING:**

Berlin Institute of Health, German Federal Ministry of Education, and German Federal Institute for Drugs and Medical Devices.

## Graphical abstract

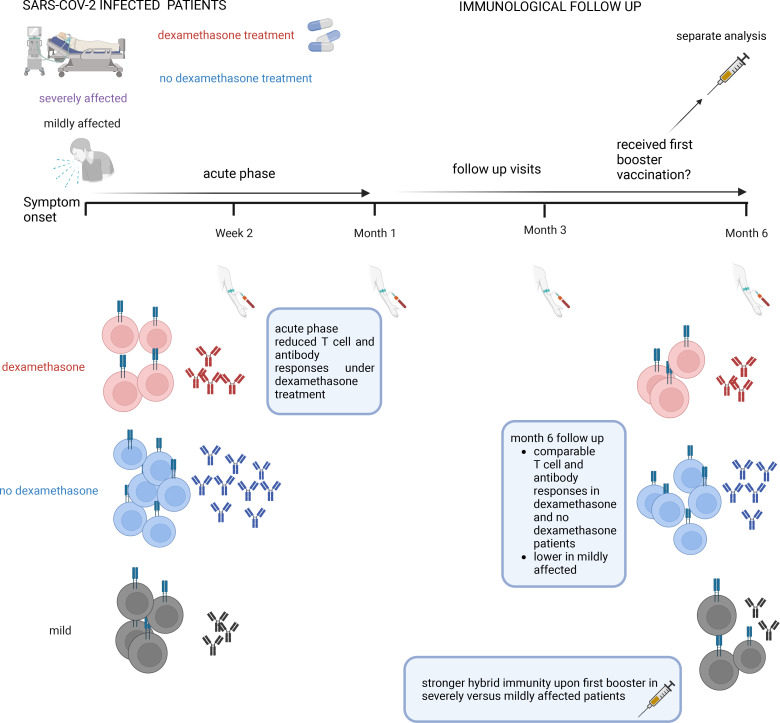

## Introduction

The clinical presentation of severe acute respiratory syndrome coronavirus type 2 (SARS-CoV-2) infection can range from asymptomatic infection to critical disease (1). Upon SARS-CoV-2 infection, the initial innate immune response is followed by T and B cell activity, including the generation of neutralizing antibodies within days after symptom onset. All of these components contribute to efficient clearance of infection and protection from severe disease in the majority of healthy individuals (2).

Among patients requiring respiratory support, dexamethasone has been shown to reduce progression to invasive mechanical ventilation and mortality at day 28 when started during the second week of the disease. To date, systemic corticosteroids remain one of the few therapeutic options available to tackle an imbalanced host immune response in severe COVID-19 that may contribute to tissue damage and subsequent organ failure (3).

SARS-CoV-2 spike–reactive B cells, CD4^+^ T cells and, to a lesser extent, CD8^+^ T cells have been described in the majority of convalescent COVID-19 patients (4), including those with mild or asymptomatic infection (5), with formation of substantial immune memory across convalescent individuals (6).

T cell responses during acute COVID-19 are multifaceted and heterogeneous across individuals (4, 7), with more pronounced T cell responses in mildly affected and younger individuals, presumably due to their larger repertoire of naive T cells, which declines with age (4–6). An early induction of T cell immunity is associated with protection from severe disease (2, 4, 8). Recent studies also highlighted several pathological immunotypes in severely affected patients, characterized for instance by a dysfunctional myeloid cell phenotype (9), a complement-mediated overactive T cell response with excessive cytotoxicity (10), or T cell exhaustion and dysfunction, resulting in insufficient infection control (5, 11). On the accompanying B cell arm, the humoral immune response to SARS-CoV-2 typically includes IgM and IgA in the first weeks after symptom onset, followed by longer-lived IgG antibody responses (2, 12). SARS-CoV-2 antibody titers have been shown to directly correlate with neutralizing serum activity in several studies (12, 13). Neutralizing activity after infection is highly variable and higher levels of neutralizing antibodies are associated with older age and more severe disease (6, 12, 14–16).

As the SARS-CoV-2 spike is highly immunogenic for T and B cells, most COVID-19 vaccines currently authorized are based on the spike protein or its subunits (17), rendering spike-reactive immune responses of particular interest when studying long-term immunity to SARS-CoV-2 infection or vaccination. Given the importance of immune memory for protection against reinfection, longitudinal studies of COVID-19 immunity have been a research priority since the beginning of the pandemic. IgG memory B cell levels remain high even 8 months after symptom onset (16) and demonstrate continuous clonal evolution (18, 19), while antibody levels in the plasma wane in this timeframe (12, 18, 19). The half-life of neutralizing IgG antibodies was estimated to be approximately 8 months (12, 20), and occurrence of breakthrough infections correlated with low levels of neutralizing antibodies (21), making them an important correlate of protection (22). The importance of neutralizing antibodies is underlined by the emergence of variants of concern with immune escape such as B.1.617.2 (Delta) and the Omicron strain sublineages (23–25), resulting in breakthrough infections in a substantial proportion of recovered and/or vaccinated individuals (26).

Several studies demonstrated that immunity after 1 natural infection leads to insufficient protection from severe COVID-19, particularly with regard to variants of concern with immune-evasive properties (27, 28). Therefore, booster immunization after natural infection is recommended and known to induce potent and broad T and B cell immunity, including neutralizing antibodies reactive against several SARS-CoV-2 variants of concern (29–31), a phenomenon termed “hybrid immunity” (32).

Long-term immunity may also be affected by treatment during the acute phase. Besides antiviral treatment, immunotherapeutic agents are a pillar of COVID-19 therapy (33). Dexamethasone has been adopted as standard-of-care in the majority of hospitalized, severely affected COVID-19 patients since mid-June 2020 (34). To date, large numbers of convalescent individuals worldwide have been treated with this corticosteroid during acute infection. We and others have previously described comparable virus shedding and antibody kinetics in severely affected patients treated with and without dexamethasone during the acute phase (35–38), but comprehensive knowledge of the differential effect of this immunosuppressive treatment on long-term and hybrid immunity, including protection against SARS-CoV-2 variants, is still scarce.

In this work, we specifically focused on comparing 3 groups of patients with (a) mild to moderate COVID-19; (b) severe COVID-19 before introduction of dexamethasone as standard-of-care, i.e., without dexamethasone treatment; and (c) severe COVID-19 treated with dexamethasone. We aimed to investigate SARS-CoV-2 spike–reactive T cells, quantitative antibody responses, and virus neutralization capacity against the Alpha and Delta variants of concern longitudinally from the acute phase to up to 6 months after symptom onset. While all samples until followup at month 6 were taken from patients without any vaccination, we additionally also investigated differences in immunological response to first booster immunization after infection in these 3 patient groups in a separate analysis.

## Results

### Clinical characteristics.

**Figure 1:**
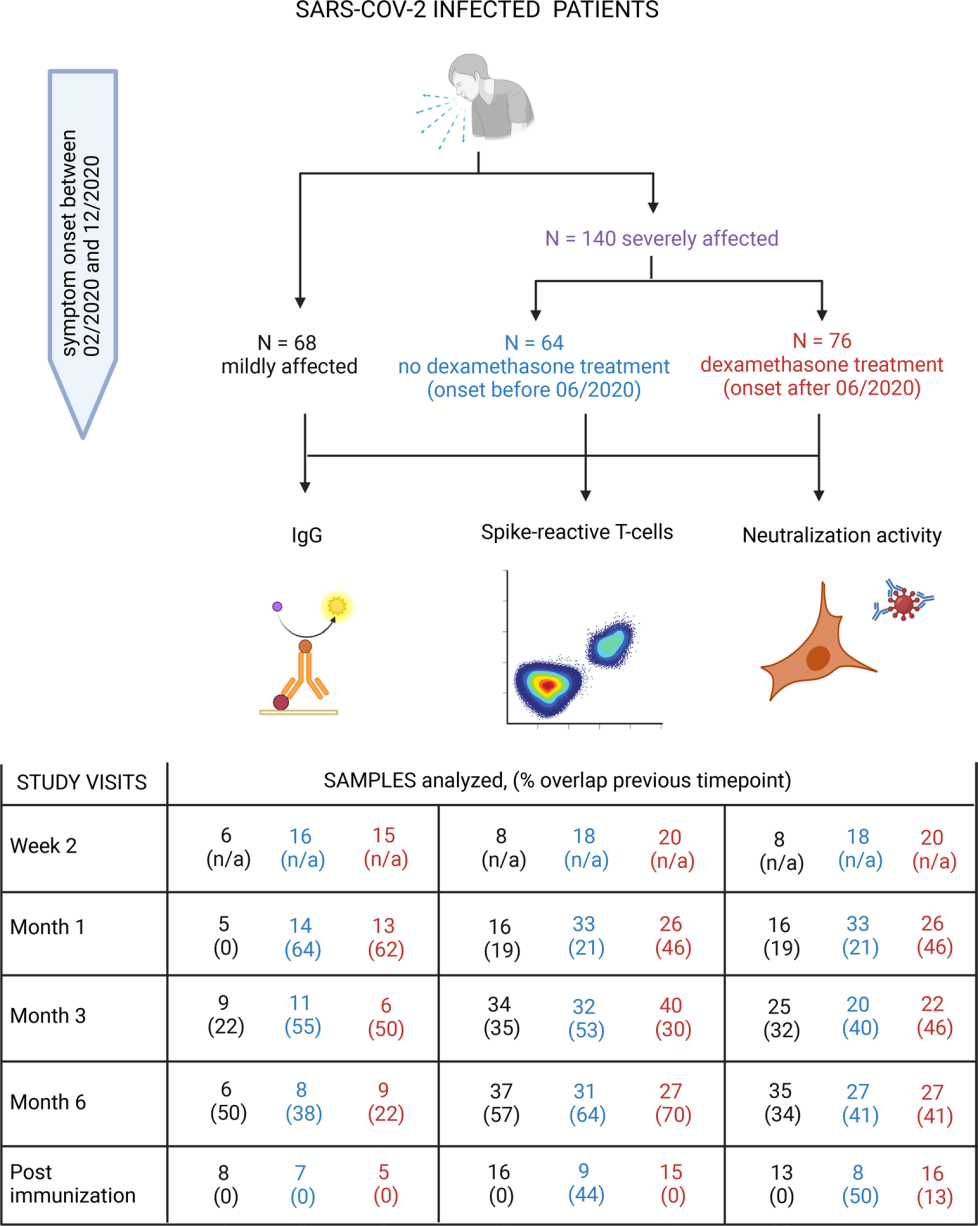
Study flow chart. A total of 208 patients with confirmed SARS-CoV-2 infection and symptom onset between February and December 2020, from prospective observational studies conducted at Charité Universitaetsmedizin Berlin, Germany, were included in this analysis. Sixty-eight were mildly affected and 140 severely affected. Of the severely affected, 64 were treated without and 76 were treated with dexamethasone within standard-of-care according to their date of disease onset. Spike-reactive T cells, RBD IgG titers, and neutralization activity against B.1.1.7 and B.1.6.172 and for postimmunization against BA.2 were measured in blood samples obtained at week 2, month 1, month 3, month 6 after symptom onset, and after single immunization. Numbers of individuals and the proportional overlap with the respective previous time point for each measurement, study visit, and group are shown in the table. N/A, not applicable for the first visit.

A total of 208 immunocompetent COVID-19 patients with symptom onset between February 2020 and December 2020 were included at Charité - Universitaetsmedizin Berlin, Germany, i.e., patients all infected in the first and second wave with SARS-CoV-2 wild type before the detection of variants of concern in Germany. Of those, 140 patients were severely affected by COVID-19 (median max. WHO score 7, IQR 4–7) and 68 were mildly affected (median max. WHO score 2, IQR 2–3). Among patients with severe disease course, 64 were hospitalized before June 2020 and did not receive dexamethasone treatment (D^–^), whereas 76 patients hospitalized thereafter received dexamethasone treatment (D^+^) of 6 mg/day orally or intravenously for a median duration of 9 days (IQR 8–10), initiated at a median of 8 days (IQR 6–11) after symptom onset. The study flow chart and details on the overlap of individuals between study visits are shown in Figure 1. Severely affected patients were older than those with mild infection, predominantly male, and had a higher weighted comorbidity index. D^+^ and D^–^ patients were of comparable age, sex distribution, and Charlson comorbidity index. The proportion of deceased patients was slightly lower in D^+^ versus D^–^ patients, without reaching statistical significance, and there were no deaths in the group of patients with mild to moderate COVID-19 (see Table 1).

### Dexamethasone-treated COVID-19 patients have a different evolution of inflammatory markers as compared with nontreated patients.

**Figure 2:**
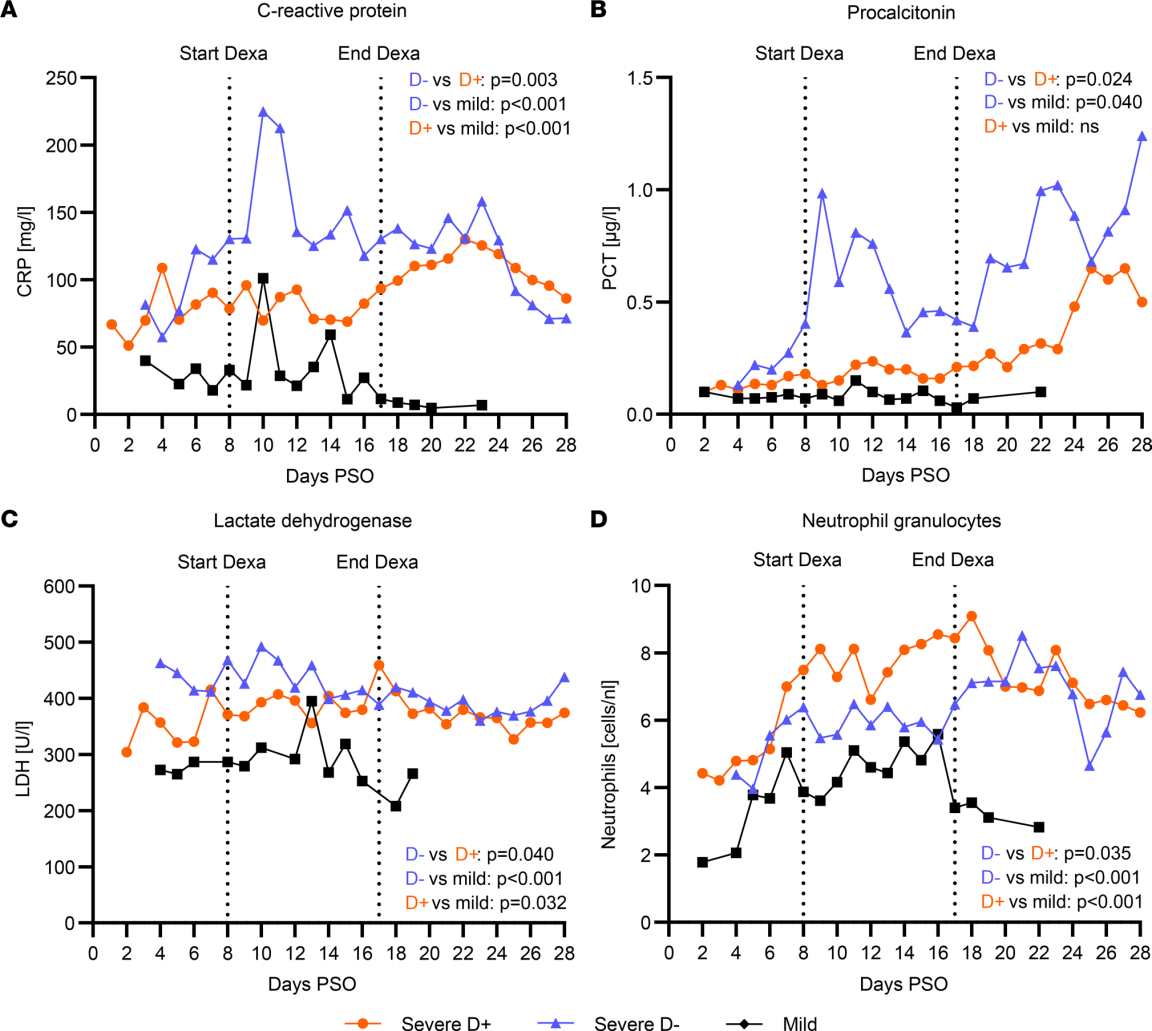
Courses of standard laboratory values during the acute phase are different in severely affected COVID-19 patients with and without dexamethasone treatment and mildly affected patients. Standard laboratory values C-reactive protein (CRP) (**A**), procalcitonin (PCT) (**B**), lactate dehydrogenase (LDH) (**C**), and absolute counts of neutrophil granulocytes (**D**) over time in mildly affected patients (black) and severely affected patients treated with dexamethasone (D^+^, red) and without dexamethasone (D^–^, blue) from symptom onset until day 28 post symptom onset (PSO) are shown. Medians of at least 3 individuals per time point are plotted. Dotted lines indicate median start (day 8 PSO) and end (day 17 PSO) of dexamethasone treatment in the D^+^ group. Slopes of the standard laboratory values were compared using mixed linear models for longitudinal comparison.

In order to analyze the potential impact of dexamethasone on the inflammatory response during the acute phase in our patient groups, we first compared the course of standard laboratory parameters known to be associated with disease severity in COVID-19, including C-reactive protein (CRP), procalcitonin (PCT), lactate dehydrogenase (LDH), and total neutrophil granulocyte counts (Figure 2 and Supplemental Figure 1; supplemental material available online with this article; https://doi.org/10.1172/jci.insight.166711DS1) as well as IL-6, ferritin, total leucocyte counts, and total lymphocyte counts (Supplemental Figure 2) from day 1 until day 28 after symptom onset. Using linear mixed models, we found a significantly different evolution of inflammatory markers in D^+^ when compared with D^–^ patients for CRP (*P* = 0.003), PCT (*P* = 0.024), LDH (*P* = 0.041), IL-6 (*P* = 0.009), and neutrophils (*P* = 0.035). As expected, mildly affected patients had different slopes of inflammatory markers over time as compared with D^+^ (Figure 2 and Supplemental Figures 1 and 2).

### Dexamethasone-treated patients have lower frequencies of SARS-COV-2 spike–reactive T cells than nontreated patients during the acute phase of infection but not at 6-month followup.

**Figure 3:**
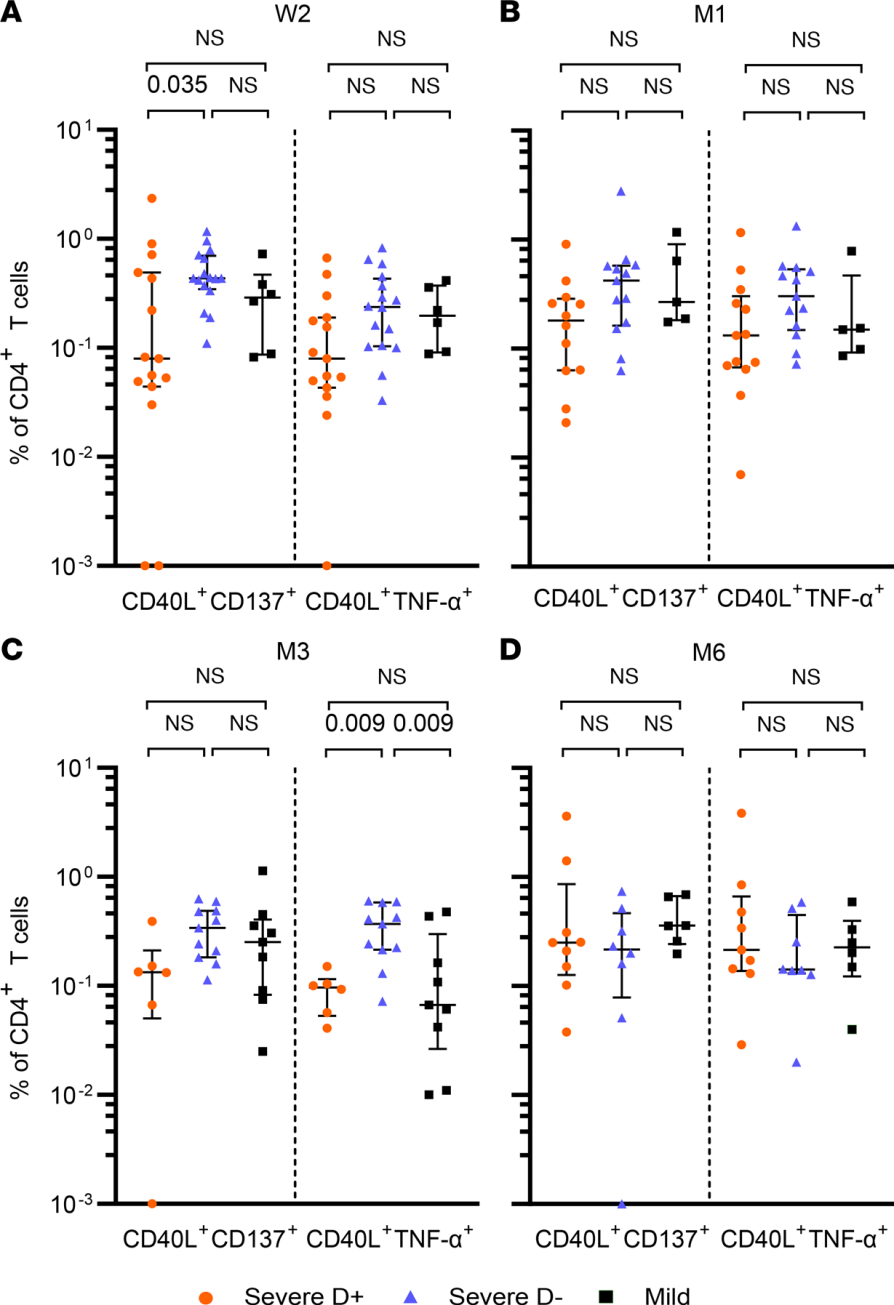
Frequencies of spike-reactive T cells are different in severely affected COVID-19 patients with and without dexamethasone treatment and mildly affected patients. Frequencies of CD40L^+^CD137^+^ and CD40L^+^TNF-α^+^ cells as percentage of CD4^+^ T cells after stimulation with a SARS-CoV-2 spike S1 peptide pool are shown. CD4^+^ T cells were measured in severe COVID-19 patients treated with dexamethasone (D^+^, red) or without (D^–^, blue) and mildly affected patients (black) at week 2 (**A**), month 1 (**B**), month 3 (**C**), and month 6 (**D**) after symptom onset. Medians and IQR are shown. Statistical testing was performed using Kruskal-Wallis test with Conover’s test for multiple comparisons.

To detect SARS-CoV-2 spike–specific T cells, PBMCs were stimulated with a SARS-COV-2 spike S1 peptide pool and stained for activation markers and cytokines. When comparing CD4^+^ T cells after stimulation, we observed significantly lower frequencies of CD40L^+^CD137^+^CD4^+^ T cells in D^+^ versus D^–^ patients in the acute phase of infection under dexamethasone treatment at week 2 (Figure 3A and Supplemental Table 1). A similar trend was observed for all other marker combinations, although not reaching statistical significance (Figure 3A and Supplemental Figure 4A). These lower frequencies of reactive CD4^+^ T cells in D^+^ patients were further observed for all marker combinations at month 1 and month 3, i.e., after the end of dexamethasone treatment, with differences for CD40L^+^IL-21^+^CD4^+^ T cells at month 1 and both CD40L^+^TNF-α^+^CD4^+^ and CD40L^+^IFN-γ^+^CD4^+^ T cells at month 3 reaching statistical significance (Figure 3C, Supplemental Figure 4A, and Supplemental Table 1). At month 6, the differences between treated and untreated patient groups had evened out and there were similar frequencies of CD4^+^ T cell subsets in D^+^ and D^–^ patients (Figure 3D and Supplemental Figure 4A).

Further, we observed lower frequencies of S1-reactive CD40L^+^TNF-α^+^CD4^+^ T cells in mildly affected compared with D^–^ patients at month 3 (Figure 3C and Supplemental Table 1) and a trend for most marker combinations between week 2 and month 3 (Figure 3, A and B, Supplemental Figure 4A, and Supplemental Tables 1 and 2). The differences between the mildly affected and D^–^ groups evened out at month 6 (Figure 3D and Supplemental Figure 4A).

When comparing CD8^+^ T cells upon S1 stimulation, there were no significant differences between D^+^ and D^–^ patients across all time points and no trend was observed. Mildly affected patients showed a trend toward higher frequencies of spike-reactive CD8^+^ T cells in comparison with both D^–^ and D^+^ at week 2 and month 3 (Supplemental Figure 5A).

In summary, we found lower CD4^+^ spike-reactive T cell frequencies under dexamethasone treatment or shortly after, but these differences evened out 6 months after symptom onset. Mildly affected patients had lower reactive CD4^+^ T cell frequencies than severely affected COVID-19 patients without dexamethasone treatment.

### Early, but not long-term, IgG response and neutralizing activity are impaired in dexamethasone-treated severe COVID-19 patients.

**Figure 4:**
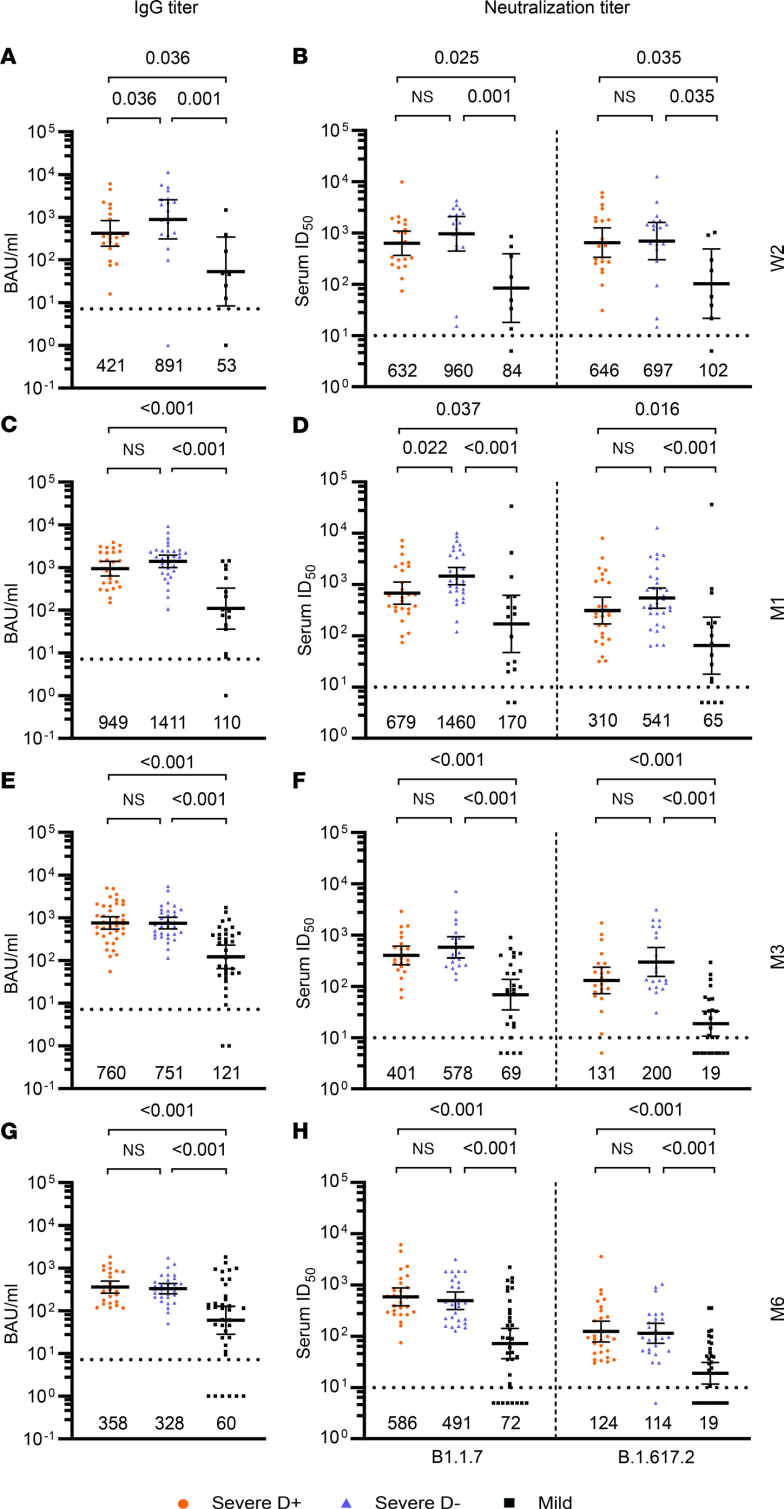
Early, but not long-term, antibody responses are different in severely affected COVID-19 patients with and without dexamethasone treatment. RBD-binding IgG titers (**A**, **C**, **E**, and **G**) and serum neutralization activity depicted as ID_50_ titers in pseudovirus neutralization testing against B.1.1.7 and B.1.617.2 at week 2, month 1, month 3, and month 6 after symptom onset (**B**, **D**, **F**, and **H**) in mildly affected patients (black) and severely affected treated with dexamethasone (D^+^, red) and without dexamethasone (D^–^, blue). Geometric means with 95% CI are shown. Black numbers indicate geometric means. Statistical testing was performed using Kruskal-Wallis test with Conover’s test for multiple comparisons. Dotted lines indicate cutoff for reactivity or neutralization.

To study the adaptive humoral immune response following infection and dexamethasone treatment, we first quantified receptor binding domain–binding (RBD-binding) IgG titers. The group of D^+^ patients showed lower median titers than D^–^ patients at week 2, i.e., during acute infection (Figure 4A and Supplemental Table 1). However, no difference in IgG titers between the 2 groups were observed at later time points between 1 and 6 months after symptom onset. Mildly affected patients showed significantly lower median RBD IgG levels in comparison with D^–^ and also D^+^ patients at all time points (Figure 4, A, C, E, and G, and Supplemental Table 1). Of note, at 6 months after symptom onset, 18.9% (7/37) of all convalescent patients after mild to moderate infection showed IgG titers below the cutoff for reactivity (Figure 4G).

In order to compare the virus-neutralizing activity between the 3 groups, serum neutralization was measured against the B.1.1.7 and B.1.617.2 spike proteins in a lentivirus-based pseudotype assay. Consistently, D^+^ patients had significantly lower 50% serum inhibitory dilution (serum ID_50_) titers against B.1.1.7 early after infection at month 1 compared with D^–^ patients (Figure 4B and Supplemental Table 1). The group of D^+^ patients also showed a trend toward lower neutralizing titers against B.1.617.2 at month 1, as well as against both B.1.1.7 and B.1.617.2 at month 3 (Figure 4, B, D, and F, Supplemental Table 1), whereas no differences between both groups were observed at month 6 (Figure 4H and Supplemental Table 1).

Mildly affected patients had lower serum ID_50_ titers against both spike variants compared with both D^–^ and D^+^ patients at all time points (Figure 4, B, D, F, and H, and Supplemental Table 1). In congruence with the overall lower RBD IgG levels of the mildly affected patients at month 3, 16% (4/25) and 40% (10/25) of patients in this group had no serum neutralizing activity against B.1.1.7 and B.1.617.2, respectively (Figure 4F). At month 6, this was true for 23% (8/35) and 40% (14/35) of patients, respectively (Figure 4H).

In summary, dexamethasone treatment resulted in lower antibody levels at week 2 and lower virus-neutralization activity against B.1.1.7 (by trend also for B.1.617.2) at month 1, i.e., under dexamethasone treatment or shortly after, but these differences evened out the following months. From month 3 after symptom onset, antibody levels and neutralizing capacity were similar in the treated and untreated severe group and remained higher than those of the mildly affected patient group throughout the observation period.

### Dexamethasone-treated and untreated recovered COVID-19 patients show comparable T cell and antibody response after booster immunization, which are higher than those in mildly affected recovered patients and double-vaccinated controls.

In order to determine the effect of booster immunization after infection, we compared SARS-CoV-2 spike–reactive T cells, RBD-binding IgG titers, and serum neutralization activity against B.1.1.7, B.1.617.2, and BA.2 spike proteins after 1 dose of a COVID-19 vaccine in all 3 convalescent patient groups. Median time from symptom onset until first immunization was 204 days (IQR 180–257) for mildly affected patients, 194 days (IQR 172–242) for D^+^, and 373 days (IQR 252–379) for D^–^ patients. Median time from immunization until biosampling was 30 days (IQR 21–73) for mildly affected, 49 days (IQR 16–102) for D^+^, and 13 (IQR 7–51) for D^–^ patients. We used samples from individuals without history of SARS-CoV-2 infection vaccinated with 2 doses of an mRNA COVID-19 vaccine (Pfizer BioNTech, BNT162b2) (median time between first and second vaccination 21 days, IQR 21–21; median time between second vaccination and sampling 27 days, IQR 26–28) as controls. The control group of vaccinated individuals was comparable to pooled mildly and severely affected COVID-19 patients in age (59 years, IQR 44.5–77.7, versus 57 years, IQR 44–66; *P* = 0.257) and sex distribution (64% male, 9/14, versus 67% male, 140/208; *P* = 0.817).

**Figure 5:**
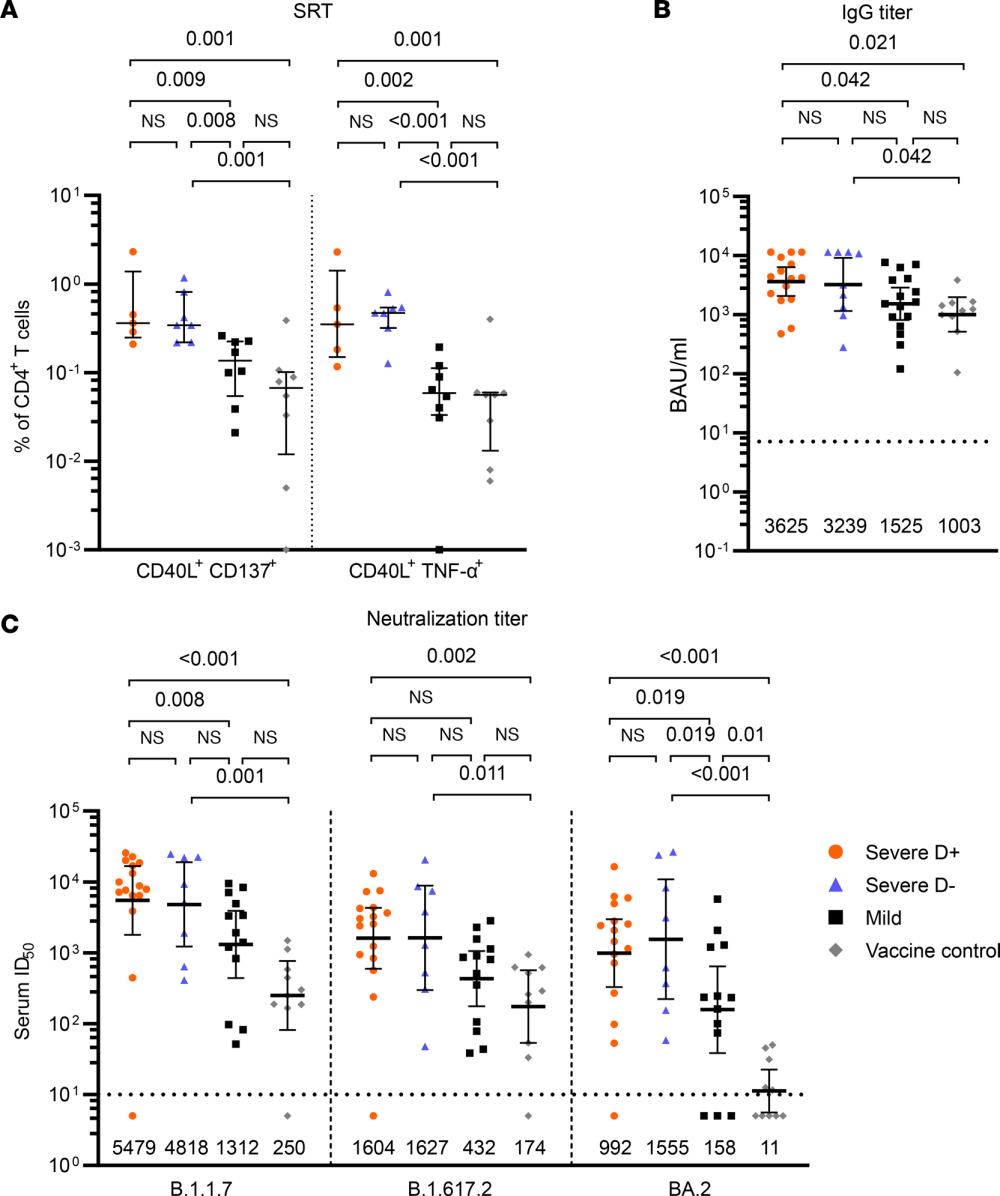
Comparable T cell and antibody response after single immunization in severely affected recovered patients with and without dexamethasone treatment in the acute phase. Comparison of spike-reactive T cells (SRT) (**A**), RBD IgG titers (**B**), and serum ID_50_ titers (**C**) in mildly affected patients (black) and severely affected patients treated with dexamethasone (D^+^, red) and without dexamethasone (D^–^, blue) during the acute phase who received a single immunization after infection and controls without history of SARS-CoV-2 who received 2 doses of mRNA vaccine (gray). **A**: Frequencies of S1-reactive CD40L^+^CD137^+^CD4^+^ and CD40L^+^TNF-α^+^CD4^+^ T cells. Medians and IQR are shown. **B**: RBD IgG titers and **C**: serum ID_50_ titers against B.1.1.7 and B.1.617.2 and BA.2. Geometric means with 95% CI are shown. Black numbers indicate geometric means, dotted lines indicate cutoff for reactivity/neutralization. Statistical testing for all panels was performed using Kruskal-Wallis test with Conover’s test for multiple comparisons.

There was no statistically significant difference in spike-reactive CD4^+^ T cells, RBD IgG, and serum ID_50_ titers between D^+^ and D^–^ patients after immunization. However, reactive CD4^+^ T cell frequencies as well as IgG and serum ID_50_ titers in both D^–^ and D^+^ patients were higher than those observed in convalescent patients after mild infection, and interestingly also compared with the double-vaccinated controls (Figure 5, Supplemental Figure 6, and Supplemental Tables 1 and 2). With the exception of 1 D^+^ patient and 1 vaccine control, all patients and vaccine controls had reactive IgG titers and neutralizing capacity against B.1.1.7 and B.1.617.2. The same D^+^ patient (6.3%, 1/16) and 23.1% (3/13) of the group of mildly affected convalescent patients and 50% (5/10) of all vaccine controls failed to neutralize BA.2 (Figure 5C).

To further investigate potential differences in postimmunization immunity in patient cohorts with different disease severity, we pooled D^+^ and D^–^ patients as “severe” and compared them to mildly affected patients and vaccine controls. Indeed, postimmunization spike-specific CD40L^+^CD137^+^CD4^+^ and CD40L^+^TNF-α^+^CD4^+^ T cell frequencies, IgG titers, and neutralization titers against all spike proteins were significantly higher in the severe group compared with the mildly affected convalescent patients and compared with the vaccine control group (Supplemental Figure 7 and Supplemental Table 2). Interestingly, there were no significant differences between convalescent patients after mild infection and the vaccine control group regarding median frequencies of spike-reactive CD4^+^ T cells and IgG titers (Figure 5, Supplemental Figure 6, and Supplemental Tables 1 and 2).

## Discussion

Severe COVID-19 is marked by a sustained proinflammatory response and subsequent tissue damage (39), potentially leading to organ failure and need for oxygen therapy or invasive mechanical ventilation and other organ replacement therapies (1). Therefore, management of SARS-CoV-2 infection in the acute phase is critical. Immunotherapeutic agents play a crucial role in the attempt to rebalance the host immune response in order to lower morbidity rates (33). Dexamethasone treatment starting on day 7 after symptom onset significantly reduced mortality in patients requiring oxygen therapy in the RECOVERY trial, with the greatest effect being shown in patients requiring mechanical ventilation (34). Dexamethasone is a long-acting, inexpensive, and readily available glucocorticoid, has a well-known safety profile, and has been in clinical use for a wide range of inflammatory conditions for decades. The immunosuppressive effect of dexamethasone is based on its binding to the glucocorticoid receptor and antagonizing transcription of proinflammatory genes (40). Yet, its long-term effect on adaptive immunity in infectious diseases such as COVID-19 is poorly understood (41). This work represents a comparative longitudinal analysis of adaptive immunity in immunocompetent severely affected patients with and without dexamethasone treatment and mildly to moderately affected COVID-19 patients based on observational data.

We observed a different evolution of CRP, PCT, IL-6, LDH, and neutrophils over time associated with dexamethasone treatment during the acute phase of the disease. Our results corroborate previous studies, which showed that dexamethasone attenuates the inflammatory response in a Syrian hamster SARS-CoV-2 infection model (42) and mediates neutrophil suppression in COVID-19 acute respiratory distress syndrome patients (43).

Our results indicate that SARS-CoV-2 spike–reactive CD4^+^ T cells are transiently lower under dexamethasone treatment and up to 3 months after infection. Glucocorticoids are known to affect T cell activation in various manners, for instance by decreasing antigen presentation in antigen-presenting cells and interfering with T cell receptor signaling and T cell polarization (40, 44, 45). Several studies suggest that the inhibitory effects of glucocorticoids especially act on Th17 and Th1 cells (40, 44, 45). In accordance with this, we saw a trend for lower frequencies of IFN-γ^+^, TNF-α^+^, and IL-21^+^ S1-reactive CD4^+^ T cells, cytokines known to be produced particularly by Th17 and Th1 cells. We also observed a different evolution of IL-6 values, a Th17 cell–promoting factor, during the acute phase in patients treated with dexamethasone. Deeper phenotyping of spike-reactive CD4^+^ T cells in patients treated with and without dexamethasone should be performed to further confirm our findings in the future. Our results suggest that the effect of dexamethasone on CD8^+^ T cells is less profound, as similar frequencies of spike-reactive CD8^+^ T cells were observed in D^+^ and D^–^ patients. These results need to be treated with caution, as the S1 peptide pool used in our study is known to elicit less-pronounced responses in CD8^+^ T cells. We further observed lower RBD-binding IgG levels and serum-neutralizing activity associated with dexamethasone treatment up to 1 month after infection.

The data thus confirm an immunosuppressive effect of dexamethasone on T cell and B cell immunity during and early after treatment in severely affected COVID-19 patients. However, considering reported overshooting immune responses in severe COVID-19 (9, 10), one could also argue that the nontreated D^–^ group shows an exaggerated T cell response, which is prohibited by dexamethasone treatment. This is supported by our data, where spike-reactive CD4^+^ T cell levels in the D^+^ group were more similar to those in the mild group. It remains to be further elaborated whether it is rather a suppressed response or prevention of an exaggerated response in the D^+^ group. Reassuringly, this difference evened out 6 months after infection.

We have previously reported that dexamethasone administration within standard-of-care does not lead to a delay until seroconversion or time until peak antibody response in COVID-19 patients using a semiquantitative S1 IgG ELISA (38). Similar results were found by another study that investigated COVID-19 patients with and without prednisolone treatment at a dose comparable to that used in our study, using the same semiquantitative S1 IgG ELISA (37). A study with 67 participants described no significant differences in quantitative S1 RBD IgG and neutralizing antibody titers at 6 and 12 months after symptom onset, but a trend toward lower neutralizing antibody titers in the subgroup of patients receiving oxygen greater than 2 L/min that were treated with steroids (46). However, there can be some heterogeneity in outcomes depending on the type and dose of corticosteroids as well as treatment duration. For example, one study described no differences in both S1 and N IgG seropositivity until 100 days after high-dose corticosteroid pulses over 3 days after adjusting for confounders (35).

We observed a different evolution of soluble markers of inflammation and tissue damage in mildly affected versus severely affected COVID-19 patients during the acute phase. Our study also shows a more short-lived adaptive immune response in mildly affected versus severely affected COVID-19 patients, which is in line with findings from previous studies (47). Remarkably, from month 3 on, 40% of mildly affected patients in our cohort failed to show neutralizing serum capacity against the Delta variant of concern. This finding may argue for a vaccination of mildly affected convalescent individuals early after acute COVID-19.

Lastly, our data add evidence to the now well-established concept of hybrid immunity (32). Our data particularly demonstrate a significantly stronger immune response to a first booster immunization in severely affected compared with mildly affected convalescent patients, and also to double-vaccinated healthy controls. This boost in neutralizing antibody levels was similar for patients with or without dexamethasone treatment during infection, again supporting that dexamethasone treatment does not affect long-term B and T cell immunity to SARS-CoV-2.

Our study has several limitations. All analyses are based on observational studies. A complete followup of all patients was not possible; thus, there was considerable variability of individuals across followup time points, making data subject to interindividual differences in T cell and antibody results. Lastly, some groups had limited sample sizes, especially for the time point after immunization.

In conclusion, we show that dexamethasone treatment for severe COVID-19 affects short-term adaptive immunity, while long-term immunity remains unaffected. After a first booster immunization in convalescent patients, no effect of prior dexamethasone treatment on adaptive immune responses could be found, a reassuring finding of importance for vaccination recommendations.

## Methods

### Study cohort.

Clinical data, standard laboratory values, and biosamples were obtained from adult individuals included in 4 prospective observational cohort studies: the Pa-COVID-19 study cohort for patients presenting to the hospital or outpatient clinic with a positive SARS-CoV-2 PCR, the COVIMMUN study as a prospective health-care-worker cohort aiming at identifying risks for infection with COVID-19, and from 2 prospective cohort studies investigating safety and immunogenicity of COVID-19 vaccines: EICOV for health-care workers and COVIMMUNIZE for persons 70 years and older. All studies were conducted at Charité Universitätsmedizin Berlin from March 2020 on and by the same study team. Overlapping case report files and questionnaires were used and sample collection, processing, and biobanking were identical. Clinical data were documented as described previously (48). For all included individuals with COVID-19, symptom onset was registered according to self-reporting of patients or close-contact persons. Information on racial and ethnic categories were incomplete and could not be analyzed. Charlson comorbidity index (49) was documented for all patients with available data and WHO score for clinical improvement (50) was documented throughout hospital stay for hospitalized patients and according to self-reporting in nonhospitalized patients. A maximum WHO score of 3 or lower was defined as mild to moderate and a maximum WHO score of 4 or higher as severe disease.

All severely affected patients with available biosamples from at least 1 visit from the Pa-COVID-19 study cohort were included. The protocol and cohort have been described elsewhere (48, 51). The study is registered in the German and WHO international clinical trials registry (DRKS00021688). Patients were treated according to national guidelines: treatment with 6 mg dexamethasone orally or intravenously was introduced as standard-of-care shortly after the press release of the RECOVERY trial on June 16, 2020, in patients fulfilling the criteria of 7 or more days of symptom duration and requiring at least supplemental oxygen therapy (i.e., WHO score ≥ 4). Throughout the hospitalization period, patients received regular study visits and outpatients were invited to followup visits at week 6, month 3, and month 6 after symptom onset in our outpatient department. Healthcare workers and vaccine cohort participants were invited to contact the study team upon a positive SARS-CoV-2 PCR for sampling. Participants of the COVIMMUN study were invited to followup visits between 6 and 12 months after inclusion. Participants of the vaccination cohorts were enrolled before the first vaccination, and followup visits were conducted 3–4 weeks after the first and second vaccination, respectively. Patients with mild infection were included from the Pa-COVID cohort as well as from healthcare worker cohorts (EICOV study and COVIMMUN study) based on self-reporting of infection upon a positive test or at followup visits.

We excluded individuals with immunosuppression (at least one of the following: immunodeficiency, immunosuppressive therapy [prednisolone equivalent of ≥ 5 mg/day and/or any other immunosuppressive agents] within 90 days, chemotherapy within 90 days, history of organ transplant, HIV infection, active lymphoma, or leukemia) prior to onset of symptoms and severely affected patients having received doses of dexamethasone or other glucocorticoids not equivalent to 6 mg dexamethasone/day.

### Booster immunization.

Booster immunizations were recommended in Germany for recovered patients beginning at the end of March, 2021. Sixteen mildly and 25 severely affected (16 D^+^ and 9 D^–^) patients had already received a first booster immunization when presenting to followup visits. Of these 41 patients, 40 had received 1 dose of an mRNA vaccine and 1 had received a dose of vector-based vaccine at the time of biosampling. Participants enrolled in the EICOV and COVIMMUNIZE studies without history of SARS-CoV-2 infection, negative S1 antibodies at enrollment, negative anti-nucleocapsid antibodies during followup, and with comparable age and sex distribution as the convalescent patients who were included as vaccine controls.

### Standard laboratory data.

All standard laboratory analyses were conducted according to standard-of-care in accredited laboratories at Charité Universitätsmedizin Berlin and extracted from patient files for further analyses. Values below the lower limit of detection (LLD) for CRP (LLD 5 mg/dL), IL-6 (LLD 1.5 ng/L), and PCT (LLD 0.5 μg/L) were set to half the value of the LLD. Medians of all patients with available laboratory measurements of the respective day were plotted, and values were excluded in cases of fewer than 3 replicates.

### Biosampling.

Biosampling was performed within the study protocols at inclusion and at regular study and followup visits. Serum samples were centrifuged at room temperature and stored undiluted in aliquots at –80°C until further use. PBMCs were isolated from heparinized whole blood by density gradient centrifugation. PBMCs were frozen in cryopreservation medium and stored at –150°C until further use.

Samples taken in defined timeframes after symptom onset were included for further analyses: (a) at “week 2” (W2), ≥6 and ≤18 days, i.e., under dexamethasone treatment where applicable; (b) at “month 1” (M1), >18 and ≤60 days, i.e.; shortly after dexamethasone treatment; (c) during followup visits at “month 3” (M3), >60 and ≤140 days; and (d) “after month 6” (M6), >140 days after symptom onset. If patients had received a booster vaccination at followup visit, respective samples were excluded from analyses of followup time points and included in the postimmunization subgroup.

For vaccine controls, samples of vaccinated participants with no history of COVID-19 obtained 20–30 days after the second dose of vaccination with BNT162b2 were included.

### T cell stimulation.

Frozen PBMCs were thawed in prewarmed medium (RPMI 1640 [Gibco], 2% FCS [Sigma-Aldrich], and 0.01% Pierce Universal Nuclease [Thermo Fisher Scientific]) on the day of analysis. A total of 200,000 cells (2 × 10^6^/mL) were seeded per well in complete medium (RPMI 1640, 10% heat-inactivated human AB serum [Pan Biotech], and 1% penicillin/streptomycin [Sigma-Aldrich]) in 96-well round-bottom plates (1–4 replicates per stimulation condition, depending on cell counts but to keep conditions within wells constant) and rested for 4 hours at 37°C and 5% CO_2_. Cells were then stimulated with 1 μg/mL PepMix SARS-CoV-2 spike glycoprotein pool S1 (JPT) and anti-CD28 (Ultra-LEAF purified anti–human CD28 antibody, Biolegend, 302934). For stimulation controls, cells were incubated with DMSO only (unstimulated) or 1 μg/mL staphylococcal enterotoxin B (SEB) (Sigma-Aldrich) only. All conditions were incubated for 16 hours at 37°C and 5% CO_2_, and Brefeldin A (eBioscience, 00-4506-51) was added after 2 hours.

### Flow cytometry.

For flow cytometry staining, cells of replicate wells were pooled and washed with PBS. Cells were incubated with Zombie UV live/dead stain (BioLegend) and Trustain human FcX (BioLegend) for 15 minutes at 4°C and washed with FACS buffer (PBS, 2% FCS, 1 mM EDTA). Cells were then permeabilized using an eBioscience Foxp3/Transcription Factor Staining Buffer Set (Invitrogen) for 30 minutes at room temperature. Intracellular staining with fluorochrome-coupled antibodies was performed for all markers in titrated optimal concentrations (CD3-BV605 [clone UCHT1], CD4–APC-Fire750 [clone RPA-T4], CD8-AF700 [clone RPA-T8], CD137/4-1BB–BV421 [clone 4B4-1], CD154/CD40L-PE [clone 24-31], granzyme B–FITC [clone GB11], IFN-γ–PE-Cy7 [clone 4S.B3], TNF-α–PerCP-Cy5.5 [clone Mab11] [all BioLegend], and IL-21–eFluor660 [clone 3A3-N2, eBioscience]) for 30 minutes at 4°C. All samples were measured on a CytoFLEX LX (Beckman Coulter) using the CytExpert software. Data were analyzed using FlowJo v10.8.0 (BD Biosciences).

To analyze the frequencies of reactive T cells, frequencies from the respective unstimulated samples were subtracted. If the resulting values were less than 0, they were set to 0 for the following analyses. To plot the values on a log scale, all values below 0.001 were set to 0.001, which was lower than the lowest measured frequency.

### Quantitative assessment of SARS-CoV-2 RBD-binding IgG.

SARS-CoV-2 RBD IgG titers were measured by chemiluminescent microparticle immunoassay, using the SARS-CoV-2 IgG II Quant assay (Abbott) on the Alinity i (Abbott). According to manufacturer’s recommendations, IgG titers of 7.1 binding antibody units (BAU)/mL or higher were interpreted as reactive. A titer of 11,360 BAU/mL corresponded to the upper limit of quantification. For measurements (*n* = 4) where IgG values exceeded 11,360 BAU/mL, the value was set to 11,360 BAU/mL. For measurements with IgG levels below the cutoff of 7.1 BAU/mL, the values were set to 1.0 for plotting graphs and statistical analysis.

### Serum neutralizing activity using a SARS-CoV-2 spike pseudovirus assay.

Serum neutralization was measured in serum samples using a lentivirus-based spike-pseudotyped pseudovirus neutralization assay. SARS-CoV-2 pseudovirus particles were generated by cotransfection of individual plasmids encoding HIV Tat, HIV Gag/Pol, HIV Rev, firefly luciferase followed by an IRES and ZsGreen, along with the SARS-CoV-2 spike protein into HEK 293T cells using the FuGENE 6 Transfection Reagent (Promega). Spike sequences from the B.1.1.7 (Alpha) variant, B.1.617.2 (Delta) (52) and Omicron BA.2 variant (24) were used to measure serum neutralization, as described previously (12). Briefly, serum samples were heat inactivated at 56°C for 45 minutes and 3-fold serial dilutions of serum were coincubated with pseudovirus supernatants for 1 hour at 37°C, following which HEK 293T cells expressing the SARS-CoV-2 receptor angiotensin-converting enzyme-2 (ACE-2) (53) were added. After a 48-hour incubation at 37°C and 5% CO_2_, luciferase activity was determined by addition of luciferin/lysis buffer (10 mM MgCl_2_, 0.3 mM ATP, 0.5 mM coenzyme A, 17 mM IGEPAL [all Sigma-Aldrich], and 1 mM D-luciferin [GoldBio] in Tris-HCl) and measured on a microplate reader (Berthold). The background relative light units (RLUs) of noninfected cells were subtracted and serum ID_50_ was calculated as the serum dilution resulting in a 50% reduction in RLU compared with the untreated virus controls. This was done by plotting an agonist-versus-normalized dose-response curve with variable slope in GraphPad Prism 7.0. Values below the LLD (serum ID_50_ of 10) were assigned a value of 5 for plotting of graphs and statistical analysis.

### Statistics.

Quantification and statistical analyses were conducted with JMP v15.0 (https://www.jmp.com/en_us/home.html) for clinical data and with R v4.0.5 (https://www.r-project.org/) for comparative analyses of all laboratory data. Distribution of continuous variables is shown as median and IQR or geometric mean with 95% CI as indicated, and graphs were made using GraphPad Prism 8 and Inkscape 1.2. The graphical abstract and study flow chart were created with BioRender.com.

Longitudinal analysis of standard laboratory values was conducted using linear mixed effects models with subject-specific random effects and population-specific fixed effects to compare slopes between the groups. Comparative analyses between groups for reactive T cells and IgG and serum ID_50_ titers was performed by applying unpaired Kruskal-Wallis tests with subsequent Conover post hoc tests using FDR correction for multiple comparisons and adjusted *P* values are reported. A *P* value of less than 0.05 was considered significant.

### Study approval.

All studies were approved by the local ethics committee (Pa-COVID: EA2/066/20, COVIMMUN: EA1/068/20, EICOV: EA4/245/20, COVIMMUNIZE: EA4/244/20), and conducted according to the Declaration of Helsinki and Good Clinical Practice principles (ICH 1996). Written informed consent was obtained from all participants.

## Author contributions

CT, MW, F Kurth, F Klein, and LES conceptualized the study. CT, LB, KV, VDC, PS, DH, PTL, FM, LJL, ETH, TL, FS, MM, and the Pa-COVID study group acquired data. LB, KV, VC, and FM conducted experiments. CT, LB, KV, KAE, PS, and PM analyzed data. CT, LB, KV, KAE, PS, DH, and PTL wrote the manuscript. MW, TZ, F Kurth, F Klein, and LES supervised the study. Order of co–first authors was determined by overall contribution to the study.

## Supplementary Material

Supplemental data

## Figures and Tables

**Table 1:**
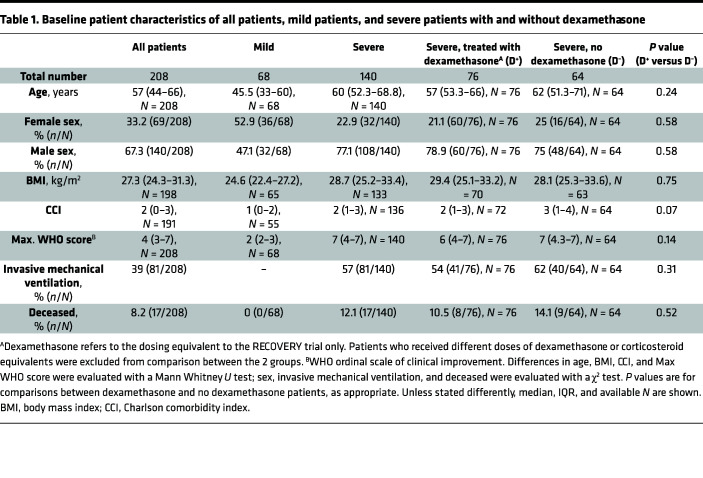
Baseline patient characteristics of all patients, mild patients, and severe patients with and without dexamethasone

## Version 1 03/07/2023

In-Press Preview

## Version 2 04/24/2023

Electronic publication

## References

[1] Hu B (2021). Characteristics of SARS-CoV-2 and COVID-19. Nat Rev Microbiol.

[2] Sette A, Crotty S (2021). Adaptive immunity to SARS-CoV-2 and COVID-19. Cell.

[3] Sterne JAC (2020). Association between administration of systemic corticosteroids and mortality among critically ill patients with COVID-19: a meta-analysis. JAMA.

[4] Rydyznski Moderbacher C (2020). Antigen-specific adaptive immunity to SARS-CoV-2 in acute COVID-19 and associations with age and disease severity. Cell.

[5] Mathew D (2020). Deep immune profiling of COVID-19 patients reveals distinct immunotypes with therapeutic implications. Science.

[6] Sekine T (2020). Robust T cell immunity in convalescent individuals with asymptomatic or mild COVID-19. Cell.

[7] Toor SM (2021). T-cell responses and therapies against SARS-CoV-2 infection. Immunology.

[8] Yu KK (2021). Comorbid illnesses are associated with altered adaptive immune responses to SARS-CoV-2. JCI Insight.

[9] Schulte-Schrepping J (2020). Severe COVID-19 is marked by a dysregulated myeloid cell compartment. Cell.

[10] Georg P (2022). Complement activation induces excessive T cell cytotoxicity in severe COVID-19. Cell.

[11] Kalfaoglu B (2020). T-cell hyperactivation and paralysis in severe COVID-19 infection revealed by single-cell analysis. Front Immunol.

[12] Vanshylla K (2021). Kinetics and correlates of the neutralizing antibody response to SARS-CoV-2 infection in humans. Cell Host Microbe.

[13] Wajnberg A (2020). Robust neutralizing antibodies to SARS-CoV-2 infection persist for months. Science.

[14] Braun J (2020). SARS-CoV-2-reactive T cells in healthy donors and patients with COVID-19. Nature.

[15] Grifoni A (2020). Targets of T cell responses to SARS-CoV-2 coronavirus in humans with COVID-19 disease and unexposed individuals. Cell.

[16] Dan JM (2021). Immunological memory to SARS-CoV-2 assessed for up to 8 months after infection. Science.

[17] Dai L, Gao GF (2021). Viral targets for vaccines against COVID-19. Nat Rev Immunol.

[18] Gaebler C (2021). Evolution of antibody immunity to SARS-CoV-2. Nature.

[19] Wang Z (2021). Naturally enhanced neutralizing breadth against SARS-CoV-2 one year after infection. Nature.

[20] Haveri A (2021). Persistence of neutralizing antibodies a year after SARS-CoV-2 infection in humans. Eur J Immunol.

[21] Bergwerk M (2021). Covid-19 breakthrough infections in vaccinated health care workers. N Engl J Med.

[22] Khoury DS (2021). Neutralizing antibody levels are highly predictive of immune protection from symptomatic SARS-CoV-2 infection. Nat Med.

[23] Gruell H (2022). mRNA booster immunization elicits potent neutralizing serum activity against the SARS-CoV-2 Omicron variant. Nat Med.

[24] Gruell H (2022). SARS-CoV-2 Omicron sublineages exhibit distinct antibody escape patterns. Cell Host Microbe.

[25] Uraki R (2022). Characterization and antiviral susceptibility of SARS-CoV-2 Omicron BA.2. Nature.

[26] Martinez-Flores D (2021). SARS-CoV-2 vaccines based on the spike glycoprotein and implications of new viral variants. Front Immunol.

[27] Nordström P (2022). Risk of SARS-CoV-2 reinfection and COVID-19 hospitalisation in individuals with natural and hybrid immunity: a retrospective, total population cohort study in Sweden. Lancet Infect Dis.

[28] Carazo S (2022). Estimated protection of prior SARS-CoV-2 infection against reinfection with the Omicron variant among messenger RNA-vaccinated and nonvaccinated individuals in Quebec, Canada. JAMA Netw Open.

[29] Reynolds CJ (2021). Prior SARS-CoV-2 infection rescues B and T cell responses to variants after first vaccine dose. Science.

[30] Stamatatos L (2021). mRNA vaccination boosts cross-variant neutralizing antibodies elicited by SARS-CoV-2 infection. Science.

[31] Goel RR (2021). Distinct antibody and memory B cell responses in SARS-CoV-2 naive and recovered individuals after mRNA vaccination. Sci Immunol.

[32] Crotty S (2021). Hybrid immunity. Science.

[33] Van de Veerdonk FL (2022). A guide to immunotherapy for COVID-19. Nat Med.

[34] Horby P (2021). Dexamethasone in hospitalized patients with Covid-19. N Engl J Med.

[35] Masiá M (2021). Lack of detrimental effect of corticosteroids on antibody responses to SARS-CoV-2 and viral clearance in patients hospitalized with COVID-19. J Infect.

[36] Chen Y, Li L (2021). Influence of corticosteroid dose on viral shedding duration in patients with COVID-19. Clin Infect Dis.

[37] Başaran S (2021). The effect of tocilizumab, anakinra and prednisolone on antibody response to SARS-CoV-2 in patients with COVID-19: a prospective cohort study with multivariate analysis of factors affecting the antibody response. Int J Infect Dis.

[38] Mühlemann B (2021). Impact of dexamethasone on SARS-CoV-2 concentration kinetics and antibody response in hospitalized COVID-19 patients: results from a prospective observational study. Clin Microbiol Infect.

[39] Delorey TM (2021). COVID-19 tissue atlases reveal SARS-CoV-2 pathology and cellular targets. Nature.

[40] Cain DW, Cidlowski JA (2017). Immune regulation by glucocorticoids. Nat Rev Immunol.

[41] Mehta J (2022). Role of dexamethasone and methylprednisolone corticosteroids in coronavirus disease 2019 hospitalized patients: a review. Front Microbiol.

[42] Wyler E (2022). Key benefits of dexamethasone and antibody treatment in COVID-19 hamster models revealed by single-cell transcriptomics. Mol Ther.

[43] Sinha S (2022). Dexamethasone modulates immature neutrophils and interferon programming in severe COVID-19. Nat Med.

[44] Elenkov IJ (2004). Glucocorticoids and the Th1/Th2 balance. Ann N Y Acad Sci.

[45] Li J (2020). Dexamethasone suppresses the Th17/1 cell polarization in the CD4^+^ T cells from patients with primary immune thrombocytopenia. Thromb Res.

[46] Epaulard O (2022). Persistence at one year of neutralizing antibodies after SARS-CoV-2 infection: influence of initial severity and steroid use. J Infect.

[47] Chia WN (2021). Dynamics of SARS-CoV-2 neutralising antibody responses and duration of immunity: a longitudinal study. Lancet Microbe.

[48] Kurth F (2020). Studying the pathophysiology of coronavirus disease 2019: a protocol for the Berlin prospective COVID-19 patient cohort (Pa-COVID-19). Infection.

[49] Charlson ME (1987). A new method of classifying prognostic comorbidity in longitudinal studies: development and validation. J Chronic Dis.

[50] https://www.who.int/docs/default-source/blue-print/covid-19-therapeutic-trial-synopsis.pdf.

[51] Thibeault C (2021). Clinical and virological characteristics of hospitalised COVID-19 patients in a German tertiary care centre during the first wave of the SARS-CoV-2 pandemic: a prospective observational study. Infection.

[52] Vanshylla K (2022). Discovery of ultrapotent broadly neutralizing antibodies from SARS-CoV-2 elite neutralizers. Cell Host Microbe.

[53] Crawford KHD (2020). Protocol and reagents for pseudotyping lentiviral particles with SARS-CoV-2 spike protein for neutralization assays. Viruses.

