# Supplementary material for: Short- and long-term T cell and antibody responses following dexamethasone treatment in COVID-19
Source: JCI Insight. 2023 Apr 24;8(8):e166711. doi: 10.1172/jci.insight.166711 (PMC10243822; doi:10.1172/jci.insight.166711)
Supplement: Supplemental data [file jciinsight-8-166711-s206.pdf]

Supplemental Table 1: Values for spike-reactive $CD4^+$ T cells ($CD40L^+$ $CD137^+$ or $CD40L^+$ TNF-α^+), RBD IgG titre and serum ID50 against B1.1.7, B1.167.2 and BA.2 in severely affected COVID-19 patients treated with dexamethasone (severe D^+) or without (severe D^-), mildly affected patients and double-vaccinated controls at week 2 (W2), month 1 (M1), month 3 (M3), month 6 (M6) and post immunization. Medians and interquartile ranges are shown.

		**time interval**				
**group**	**analysis**	**W2**	**M1**	**M3**	**M6**	**post immunization**
**severe D^+**	% $CD40L^+$ $CD137^+$ of $CD4^+$ T cells (S1)	0.08 (0.044-0.492)	0.182 (0.063-0.287)	0.133 (0.051-0.212)	0.250 (0.127-0.86)	0.362 (0.249-1.39)
	% $CD40L^+$ TNF-α^+ of $CD4^+$ T cells (S1)	0.08 (0.043-0.19)	0.132 (0.067-0.304)	0.097 (0.053-0.116)	0.214 (0.137-0.663)	0.35 (0.15-1.42)
	RBD IgG titre [BAU/ml]	343 (196-1527)	1147 (412.4-2307)	745.7 (423.1-1907)	353.1 (161.9-822.7)	4216 (1821-9383)
	serum ID_{50} B1.1.7	659.7 (257.7-1587)	577 (322.5-2254)	341 (235.1-734.6)	563.2 (295.2-1092)	8354 (6434-18155)
	serum ID_{50} B1.617.2	557.4 (259-1966)	264.1 (92.03-1178)	128.7 (71.76-280.6)	91.22 (47.03-298.7)	2791 (862.2-4335)
	serum ID_{50} BA.2	N/A	N/A	N/A	N/A	1763 (382.6-4417)
**severe D^-**	% $CD40L^+$ $CD137^+$ of $CD4^+$ T cells (S1)	0.436 (0.345-0.698)	0.423 (0.163-0.580)	0.339 (0.183-0.488)	0.216 (0.078-0.465)	0.343 (0.220-0.815)
	% $CD40L^+$ TNF-α^+ of $CD4^+$ T cells (S1)	0.238 (0.104-0.431)	0.304 (0.148-0.533)	0.369 (0.214-0.584)	0.142 (0.13-0.45)	0.473 (0.32-0.545)
	RBD IgG titre [BAU/ml]	1739 (402-3102)	1772 (799.1-2466)	674 (404.7-1452)	320.8 (210.8-528.9)	3177 (1153-11360)
	serum ID_{50} B1.1.7	1568 (565.7-2585)	1255 (735.2-3913)	439.4 (256.7-1071)	447.9 (205.6-1240)	7213 (951.8-22376)
	serum ID_{50} B1.617.2	1231 (435.4-1730)	440.7 (275.9-1204)	167.2 (101.3-1393)	96.85 (52.07-220.1)	2542 (360.6-8306)
	serum ID_{50} BA.2	N/A	N/A	N/A	N/A	1866 (208-20142)
**mild**	% $CD40L^+$ $CD137^+$ of $CD4^+$ T cells (S1)	0.290 (0.087-0.468)	0.269 (0.183-0.907)	0.253 (0.083-0.405)	0.359 (0.243-0.667)	0.137 (0.054-0.226)
	% $CD40L^+$ TNF-α^+ of $CD4^+$ T cells (S1)	0.197 (0.091-0.373)	0.150 (0.093-0.471)	0.067 (0.027-0.299)	0.226 (0.123-0.396)	0.059 (0.033-0.113)
	RBD IgG titre [BAU/ml]	47.6 (15.8-334)	99.45 (38.25-829.7)	215 (47.93-420.3)	114.7 (19.4-316.4)	1586 (704-4009)
	serum ID_{50} B1.1.7	93.81 (18.45-495.8)	253.7 (23.48-580.6)	86.71 (17.27-321.9)	90 (10.25-329.7)	1913 (464.6-6010)
	serum ID_{50} B1.617.2	121.4 (25.75-753.4)	55.57 (6.9-180.1)	15.46 (5-56.27)	21.82 (5-57.06)	809.2 (92.43-1345)
	serum ID_{50} BA.2	N/A	N/A	N/A	N/A	234.6 (39.49-1243)
**vaccine controls**	% $CD40L^+$ $CD137^+$ of $CD4^+$ T cells (S1)	N/A	N/A	N/A	N/A	0.067 (0.012-0.102)
	% $CD40L^+$ TNF-α^+ of $CD4^+$ T cells (S1)	N/A	N/A	N/A	N/A	0.056 (0.013-0.06)
	RBD IgG titre [BAU/ml]	N/A	N/A	N/A	N/A	1204 (830.8-1593)
	serum ID_{50} B1.1.7	N/A	N/A	N/A	N/A	275.8 (181-720.5)
	serum ID_{50} B1.617.2	N/A	N/A	N/A	N/A	275.3 (48.57-622.6)
	serum ID_{50} BA.2	N/A	N/A	N/A	N/A	8.29 (5-34.95)

Supplemental Table 2: Additional values for spike- and SEB-reactive CD4+ T cells (CD40L+ CD137+, CD40L+ TNF-α^{+}, CD40L^{+} IFN-γ^{+} or CD40L^{+} IL-21^{+}), spike- and SEB-reactive CD8^{+} T cells (CD137^{+} TNF-α^{+}, CD137^{+} IFN-γ^{+} or CD137^{+} GrzB^{+}), RBD IgG titre and serum ID50 against B1.1,7, B1.167.2 and BA.2 in severely infected COVID-19 patients treated with dexamethasone (severe D^{+}) or without (severe D^{-}) or both groups pooled as severe, mildly infected patients and double-vaccinated controls at week 2, month 1, month 3, month 6 and post immunization. Median and interquartile range are shown

		time interval				
group	analysis	Week 2	Month 1	Month 3	Month 6	post immunization
severe D+	% CD40L^{+} IFN-γ^{+} of CD4^{+} T cells (S1)	0.009 (0.001-0.068)	0.046 (0.015-0.149)	0.026 (0.004-0.044)	0.066 (0.026-0.237)	0.228 (0.152-0.907)
	% CD40L^{+} IL-21^{+} of CD4^{+} T cells (S1)	0.013 (0.001-0.044)	0.056 (0.013-0.096)	0.027 (0.006-0.082)	0.041 (0.008-0.137)	0.105 (0.033-0.456)
	% CD40L^{+} CD137^{+} of CD4^{+} T cells (SEB)	4.53 (3.118-9.198)	5.404 (2.786-10.48)	5.632 (2.468-10.98)	7.51 (5.586-8.126)	7.136 (5.734-8.081)
	% CD40L^{+} TNF-α^{+} of CD4^{+} T cells (SEB)	3.75 (3.029-7.664)	4.171 (1.98-9.613)	6.031 (3.661-8.781)	7.514 (5.936-8.442)	7.04 (5.72-7.475)
	% CD40L^{+} IFN-γ^{+} of CD4^{+} T cells (SEB)	1.078 (0.56-3.05)	0.866 (0.569-3.941)	1.409 (0.835-6.87)	2.776 (1.494-3.884)	2.328 (1.562-2.672)
	% CD40L^{+} IL-21^{+} of CD4^{+} T cells (SEB)	0.12 (0.06-0.32)	0.207 (0.077-0.805)	0.531 (0.06-0.863)	0.34 (0.221-0.498)	0.32 (0.297-0.468)
	% CD137^{+} TNF-α^{+} of CD8^{+} T cells (S1)	0.001 (0.001-0.144)	0.02 (0.006-0.077)	0.001 (0.001-0.027)	0.001 (0.001-0.065)	0.03 (0.001-0.58)
	% CD137^{+} IFN-γ^{+} of CD8^{+} T cells (S1)	0.001 (0.001-0.08)	0.02 (0.001-0.21)	0.001 (0.001-0.058)	0.001 (0.001-0.041)	0.02 (0.001-0.88)
	% CD137^{+} GrzB^{+} of CD8^{+} T cells (S1)	0.29 (0.001-0.54)	0.09 (0.001-0.215)	0.001 (0.001-0.215)	0.001 (0.001-0.22)	0.06 (0.001-1.335)
	% CD137+ TNF-α+ of CD8+ T cells (SEB)	4.35 (2.852-8.895)	3.92 (1.92-8.961)	9.885 (2.165-16.95)	11.54 (6.565-22.76)	6.9 (5.563-14.82)
	% CD137^{+} IFN-γ^{+} of CD8^{+} T cells (SEB)	6.587 (4.235-12.14)	5.707 (2.725-12.14)	11 (2.255-20.53)	16.05 (6.78-26.9)	6.505 (4.625-17.1)
	% CD137^{+} GrzB^{+} of CD8^{+} T cells (SEB)	6.64 (3.205-11.46)	5.81 (2.1-8.31)	10.42 (1.345-12.79)	10.01 (2.86-13.84)	4.61 (1.935-17.45)
severe D-	% CD40L^{+} IFN-γ^{+} of CD4^{+} T cells (S1)	0.086 (0.008-0.26)	0.146 (0.071-0.242)	0.16 (0.1-0.35)	0.082 (0.042-0.119)	0.353 (0.223-0.47)
	% CD40L^{+} IL-21^{+} of CD4^{+} T cells (S1)	0.044 (0.019-0.063)	0.13 (0.06-0.238)	0.106 (0.07-0.143)	0.044 (0.006-0.114)	0.096 (0.065-0.123)
	% CD40L^{+} CD137^{+} of CD4^{+} T cells (SEB)	7.33 (4.021-9.871)	8.738 (6.479-11.98)	9.799 (6.564-10.69)	9.052 (5.05-9.25)	8.57 (6.01-11.65)
	% CD40L^{+} TNF-α^{+} of CD4^{+} T cells (SEB)	5.689 (3.772-8.841)	8.218 (7.098-9.891)	8.639 (4.74-10.36)	7.26 (6.13-7.905)	8.053 (6.06-9.48)
	% CD40L^{+} IFN-γ^{+} of CD4^{+} T cells (SEB)	2.62 (1.18-4.08)	4.433 (2.056-5.997)	4.099 (1.78-6.343)	2.671 (2.222-3.411)	3.49 (1.69-4.21)
	% CD40L^{+} IL-21^{+} of CD4^{+} T cells (SEB)	0.17 (0.079-0.38)	0.36 (0.232-0.606)	0.393 (0.19-1.08)	0.517 (0.372-0.67)	0.14 (0.073-0.923)
	% CD137^{+} TNF-α^{+} of CD8^{+} T cells (S1)	0.015 (0.001-0.123)	0.001 (0.001-0.156)	0.042 (0.001-0.07)	0.001 (0.001-0.193)	0.025 (0.001-0.7)
	% CD137^{+} IFN-γ^{+} of CD8^{+} T cells (S1)	0.027 (0.003-0.275)	0.001 (0.001-0.185)	0.032 (0.001-0.23)	0.001 (0.001-0.618)	0.001 (0.001-1.14)
	% CD137^{+} GrzB^{+} of CD8^{+} T cells (S1)	0.19 (0.018-0.445)	0.03 (0.001-0.45)	0.13 (0.001-0.72)	0.001 (0.001-0.1)	0.06 (0.001-1.2)
	% CD137+ TNF-α+ of CD8+ T cells (SEB)	4.15 (1.204-6.18)	5.695 (4.392-9.8)	8.82 (6.79-10.34)	9.2 (7.89-17.01)	10.56 (8.015-14.96)
	% CD137^{+} IFN-γ^{+} of CD8^{+} T cells (SEB)	5.26 (1.95-13.52)	8.133 (5.775-15.63)	11.03 (7.74-14.18)	12.69 (9.05-21.58)	10.46 (6.272-18.57)
	% CD137^{+} GrzB^{+} of CD8^{+} T cells (SEB)	4.27 (2.7-10.8)	5.56 (3.433-12.21)	10.09 (5.35-17.6)	10.61 (7.003-14.6)	9.8 (1.91-14)

mild	% CD40L$^+$ IFN-γ$^+$ of CD4$^+$ T cells (S1)	0.026 (0.007-0.09)	0.051 (0.035-0.086)	0.021 (0.003-0.059)	0.007 (0.001-0.129)	0.019 (0.008-0.039)
	% CD40L$^+$ IL-21$^+$ of CD4$^+$ T cells (S1)	0.015 (0.007-0.081)	0.001 (0.001-0.042)	0.019 (0.005-0.045)	0.007 (0.001-0.087)	0.017 (0.005-0.032)
	% CD40L$^+$ CD137$^+$ of CD4$^+$ T cells (SEB)	5.047 (3.763-6.164)	6.266 (4.55-9.342)	5.297 (4.106-6.921)	8.859 (6.358-10.64)	3.987 (3.498-9.556)
	% CD40L$^+$ TNF-α$^+$ of CD4$^+$ T cells (SEB)	4.62 (3.426-5.434)	6.516 (3.881-8.554)	5.675 (3.79-6.634)	8.053 (5.283-10.5)	3.671 (3.425-7.381)
	% CD40L$^+$ IFN-γ$^+$ of CD4$^+$ T cells (SEB)	2.075 (1.175-3.003)	3.61 (2.343-5.608)	2.082 (1.805-3.581)	2.232 (1.056-6.485)	1.315 (0.96-2.042)
	% CD40L$^+$ IL-21$^+$ of CD4$^+$ T cells (SEB)	0.112 (0.044-0.28)	0.45 (0.384-0.494)	0.23 (0.151-0.6)	0.25 (0.068-0.348)	0.162 (0.12-0.29)
	% CD137$^+$ TNF-α$^+$ of CD8$^+$ T cells (S1)	0.21 (0.065-0.56)	0.001 (0.001-0.082)	0.15 (0.019-0.51)	0.001 (0.001-0.168)	0.145 (0.02-0.365)
	% CD137$^+$ IFN-γ$^+$ of CD8$^+$ T cells (S1)	0.35 (0.103-0.848)	0.001 (0.001-0.39)	0.47 (0.002-0.68)	0.001 (0.001-0.25)	0.11 (0.046-0.288)
	% CD137$^+$ GrzB$^+$ of CD8$^+$ T cells (S1)	0.52 (0.295-1.505)	0.001 (0.001-0.385)	0.52 (0.056-1.36)	0.086 (0.001-0.67)	0.44 (0.041-0.703)
	% CD137$^+$ TNF-α$^+$ of CD8$^+$ T cells (SEB)	7.975 (5.23-12.52)	5.03 (4.013-5.458)	6.942 (3.645-14.33)	8.92 (3.04-13.13)	6.635 (3.903-12.5)
	% CD137$^+$ IFN-γ$^+$ of CD8$^+$ T cells (SEB)	10.23 (5.865-14.97)	6.18 (4.763-8.047)	9.462 (3.46-17.47)	10.4 (3.475-13.45)	7.585 (5.128-13.53)
	% CD137$^+$ GrzB$^+$ of CD8$^+$ T cells (SEB)	9.235 (6.075-14.6)	3.825 (1.538-7.695)	5.835 (2.97-13.05)	5.555 (3-13.61)	7.985 (5.95-11.79)
vaccine controls	% CD40L+ IFN-γ+ of CD4+ T cells (S1)	N/A	N/A	N/A	N/A	0.022 (0.002-0.049)
	% CD40L$^+$ IL-21$^+$ of CD4$^+$ T cells (S1)	N/A	N/A	N/A	N/A	0.019 (0.003-0.048)
	% CD40L$^+$ CD137$^+$ of CD4$^+$ T cells (SEB)	N/A	N/A	N/A	N/A	6.484 (6.045-7.439)
	% CD40L$^+$ TNF-α$^+$ of CD4$^+$ T cells (SEB)	N/A	N/A	N/A	N/A	4.36 (3.317-7.637)
	% CD40L$^+$ IFN-γ$^+$ of CD4$^+$ T cells (SEB)	N/A	N/A	N/A	N/A	1.913 (1.163-2.085)
	% CD40L$^+$ IL-21$^+$ of CD4$^+$ T cells (SEB)	N/A	N/A	N/A	N/A	0.264 (0.208-0.378)
	% CD137$^+$ TNF-α$^+$ of CD8$^+$ T cells (S1)	N/A	N/A	N/A	N/A	0.036 (0.003-0.068)
	% CD137$^+$ IFN-γ$^+$ of CD8$^+$ T cells (S1)	N/A	N/A	N/A	N/A	0.105 (0.015-0.195)
	% CD137$^+$ GrzB$^+$ of CD8$^+$ T cells (S1)	N/A	N/A	N/A	N/A	0.205 (0.036-0.95)
	% CD137$^+$ TNF-α$^+$ of CD8$^+$ T cells (SEB)	N/A	N/A	N/A	N/A	3.65 (2.009-7.895)
	% CD137$^+$ IFN-γ$^+$ of CD8$^+$ T cells (SEB)	N/A	N/A	N/A	N/A	5.439 (2.8-9.561)
	% CD137$^+$ GrzB$^+$ of CD8$^+$ T cells (SEB)	N/A	N/A	N/A	N/A	4.417 (1.435-9.175)
pooled severe	% CD40L$^+$ CD137$^+$ of CD4$^+$ T cells (S1)	N/A	N/A	N/A	N/A	0.353 (0.237-0.724)
	% CD40L$^+$ TNF-α$^+$ of CD4$^+$ T cells (S1)	N/A	N/A	N/A	N/A	0.472 (0.217-0.544)
	RBD IgG titre [BAU/ml]	N/A	N/A	N/A	N/A	4146 (1760-11216)
	serum ID_{50} B1.1.7	N/A	N/A	N/A	N/A	8354 (4190-19664)
	serum ID_{50} B1.617.2	N/A	N/A	N/A	N/A	2791 (632.9-6576)
	serum ID_{50} BA.2	N/A	N/A	N/A	N/A	1763 (295.2-5707)

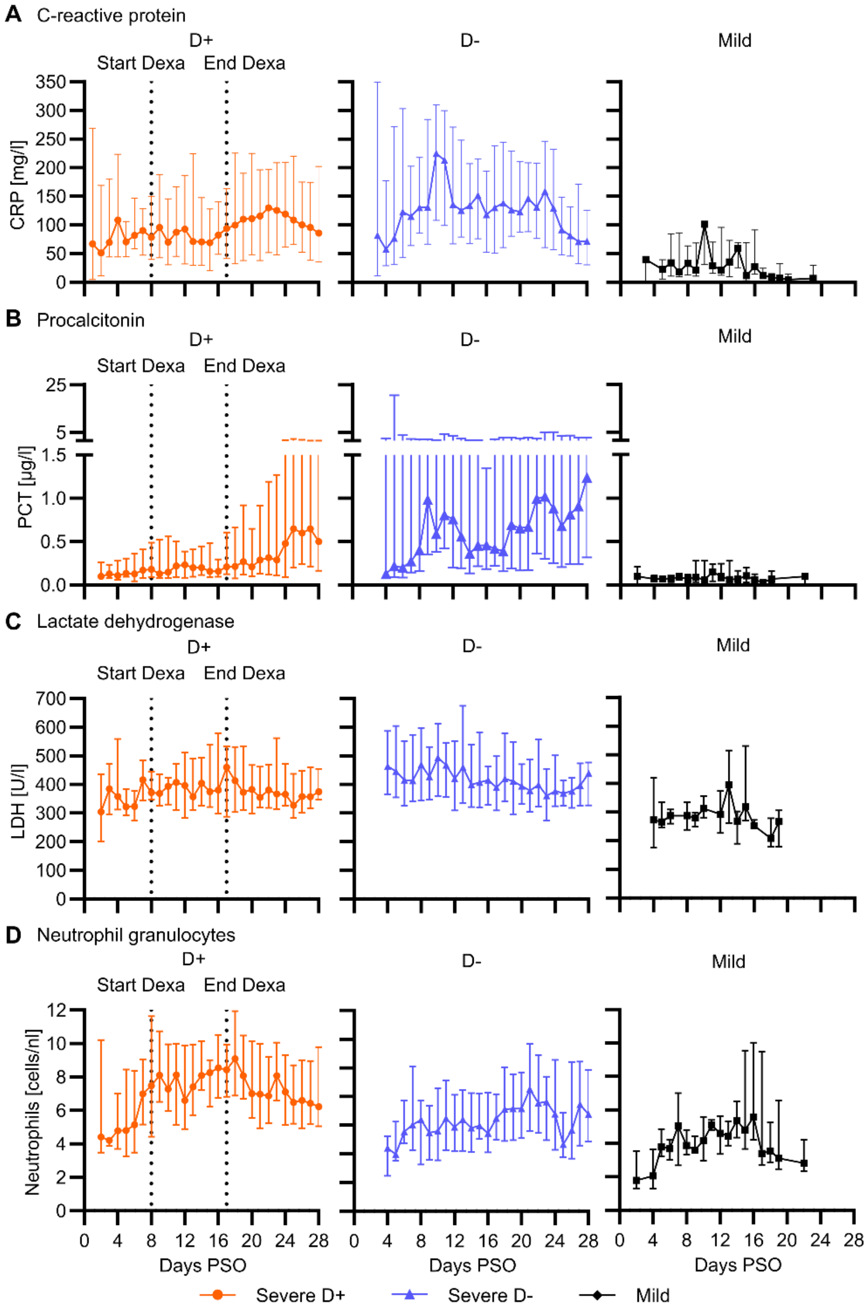

Supplemental Figure 1: Courses of standard laboratory values during the acute phase in severely affected COVID-19 patients treated with and without dexamethasone and mildly affected patients including error bars. Standard laboratory values C-reactive protein (A), Procalcitonin (B), Lactate dehydrogenase (C) and absolute neutrophil granulocytes (D) over time in mildly affected patients (black), severely affected patients treated with dexamethasone (D$^+$, red) and without dexamethasone (D$^-$, blue) from symptom onset until day 28 are shown. Medians of at least 3 individuals/timepoint and IQR are plotted. Dotted lines indicate median start (day 8 post symptom onset) and end (day 17) of dexamethasone treatment in the D$^+$ group.

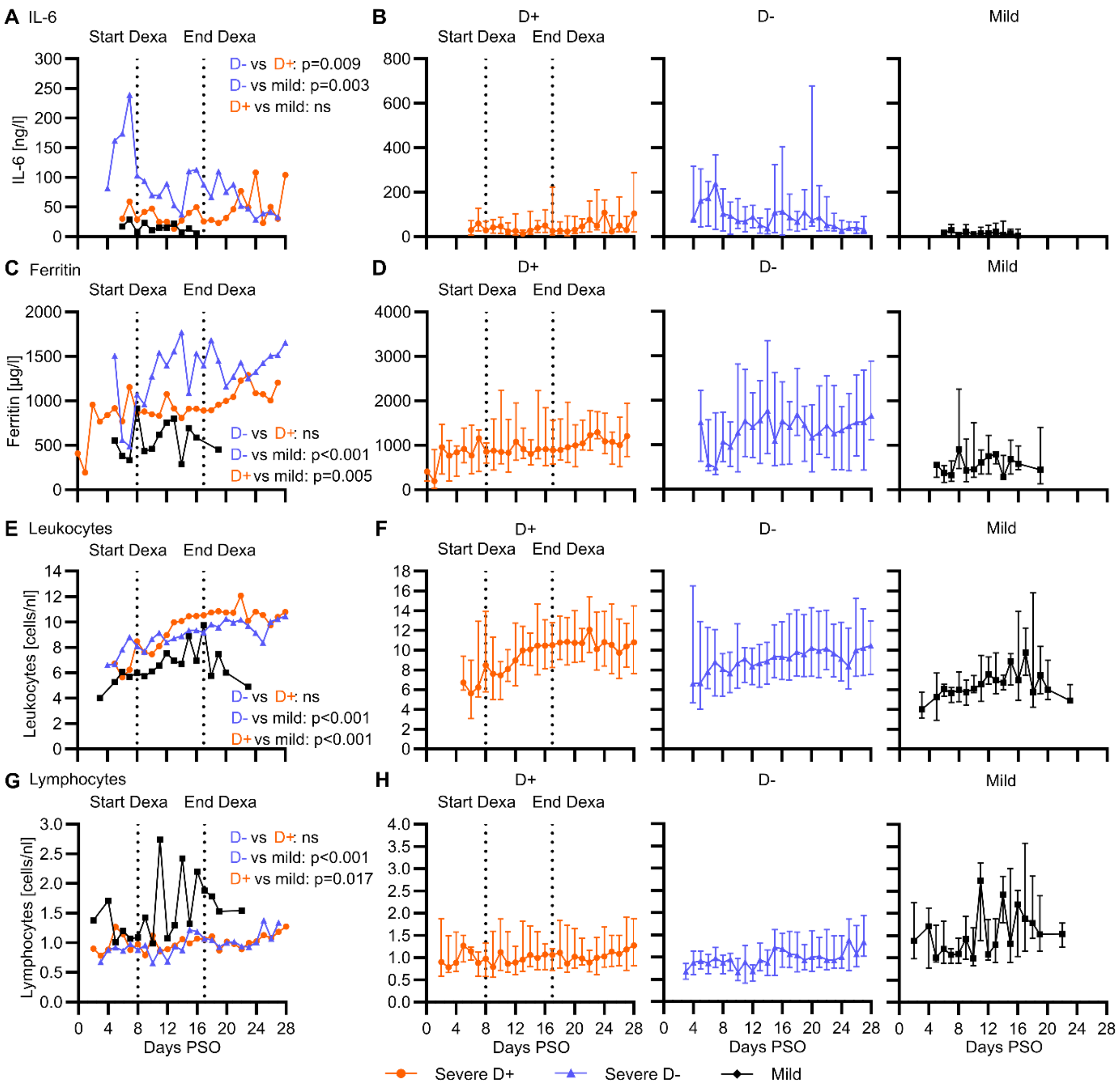

Figure S2: Courses of additional standard laboratory values during the acute phase in severely affected COVID-19 patients treated with and without dexamethasone and mildly affected patients including error bars. Values of Interleukin-6 (A), Ferritin (C), leukocytes (E) and lymphocytes (G) over time in mildly affected patients (black), severely affected patients treated with dexamethasone (D^{+}, red) and without dexamethasone (D, blue) from symptom onset until day 28 are shown. Medians of at least 3 individuals/timepoint are plotted. Panels B, D, F and H show the respective curves including IQR for better visibility. Dotted lines indicate median start (day 8 post symptom onset) and end (day 17) of dexamethasone treatment in the D^{+} group. In Panels A,C,E and G, slopes of the standard laboratory values were compared using mixed linear models for longitudinal comparison.

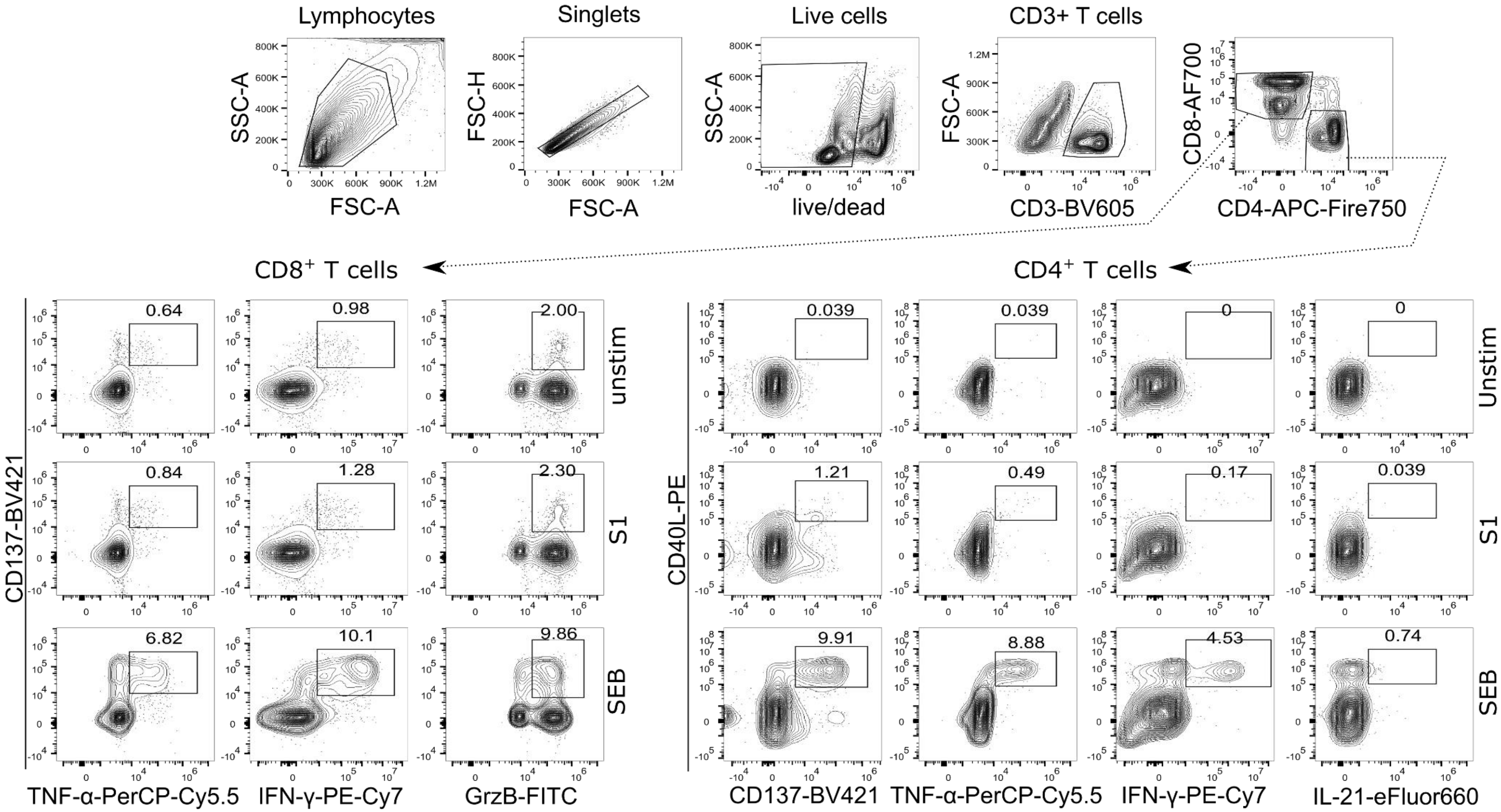

Figure S3. Gating strategy for S1-reactive CD4+ and CD8+ T cells. Contour plots representing the gating strategy for the identification of reactive CD4+ T cells and CD8+ T cells. Representative plots for unstimulated, S1- and SEB-stimulated cells of one patient of the D- group at week 2 are shown. Values indicate the percentages of positive cells.

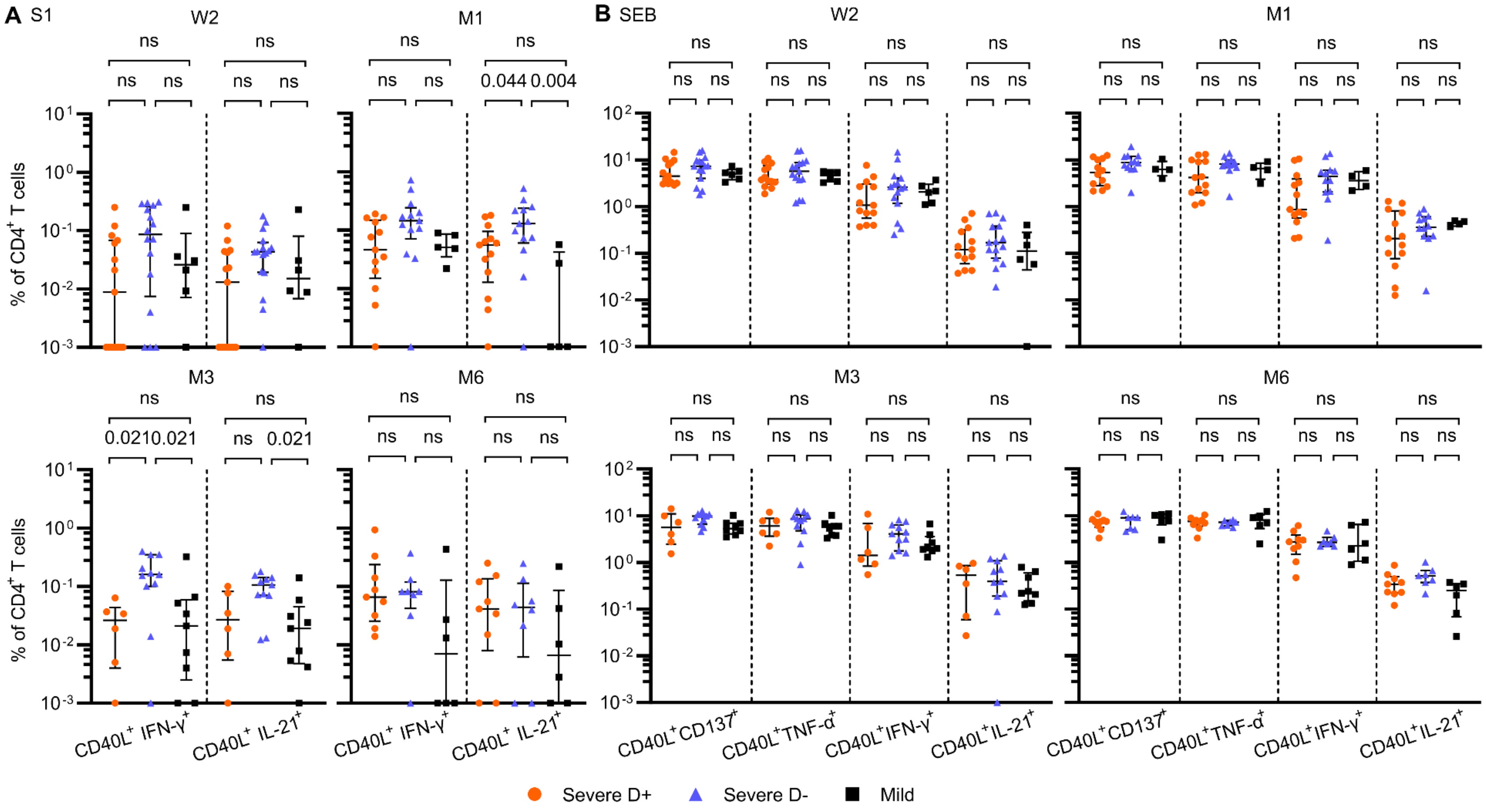

Figure S4. Frequencies of S1- and SEB-reactive CD4⁺ T cells. Reactive CD4⁺ T cells were measured in severely affected COVID-19 patients treated with dexamethasone (D⁺, red) or without (D⁻, blue) and mildly affected patients (black) at week 2, month 1, month 3 and month 6 post symptom onset. A) Frequencies of additional marker combinations for S1-reactive CD4⁺ T cells (CD40L⁺ IFN-γ⁺ and CD40L⁺ IL-21⁺) are shown as percentages of total CD4⁺ T cells. B) Frequencies of reactive CD4⁺ T cells (CD40L⁺ CD137⁺, CD40L⁺ TNF-α⁺, CD40L⁺ IFN-γ⁺ and CD40L⁺ IL-21⁺) after stimulation with SEB as positive control. Medians are shown and whiskers indicate IQR. Statistical testing was performed using Kruskal-Wallis-Test with Conover's test for multiple comparisons.

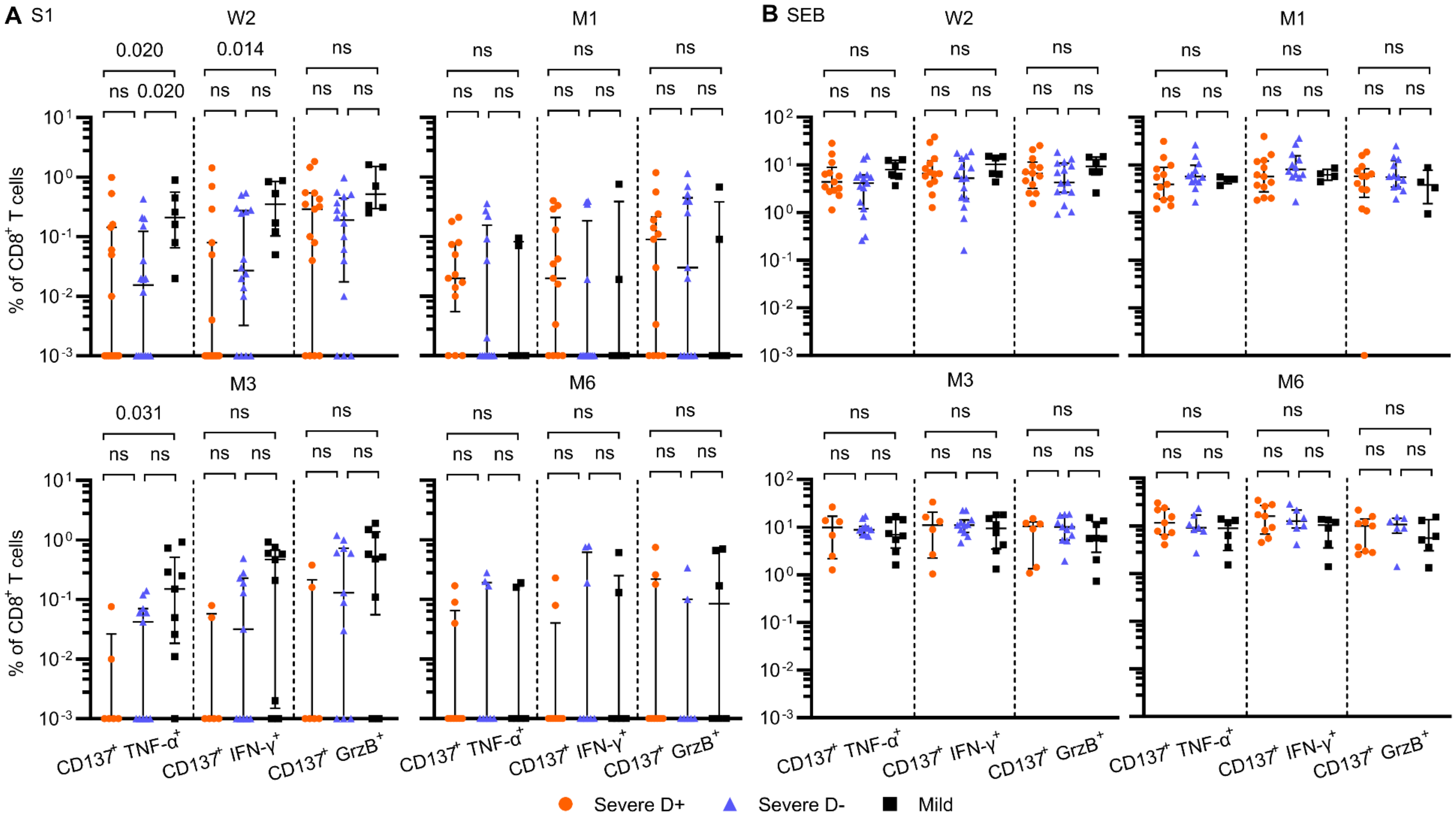

Figure S5: Frequencies of S1- and SEB-reactive CD8^{+} T cells. Reactive CD8^{+} T cells were measured in severely affected COVID-19 patients treated with dexamethasone (D^{+}, red) or without (D^{-}, blue) and mildly affected patients (black) at week 2, month 1, month 3 and month 6 PSO. Frequencies of S1- (A) and SEB-reactive (B) CD8^{+} T cells (CD137^{+} IFN-γ^{+}, CD137^{+} TNF-α^{+} and CD137^{+} GrzB^{+}) are shown as percentages of total CD8^{+} T cells. Medians are shown and whiskers indicate IQR. Statistical testing was performedusing Kruskal-Wallis test with Conover test for multiple comparisons.

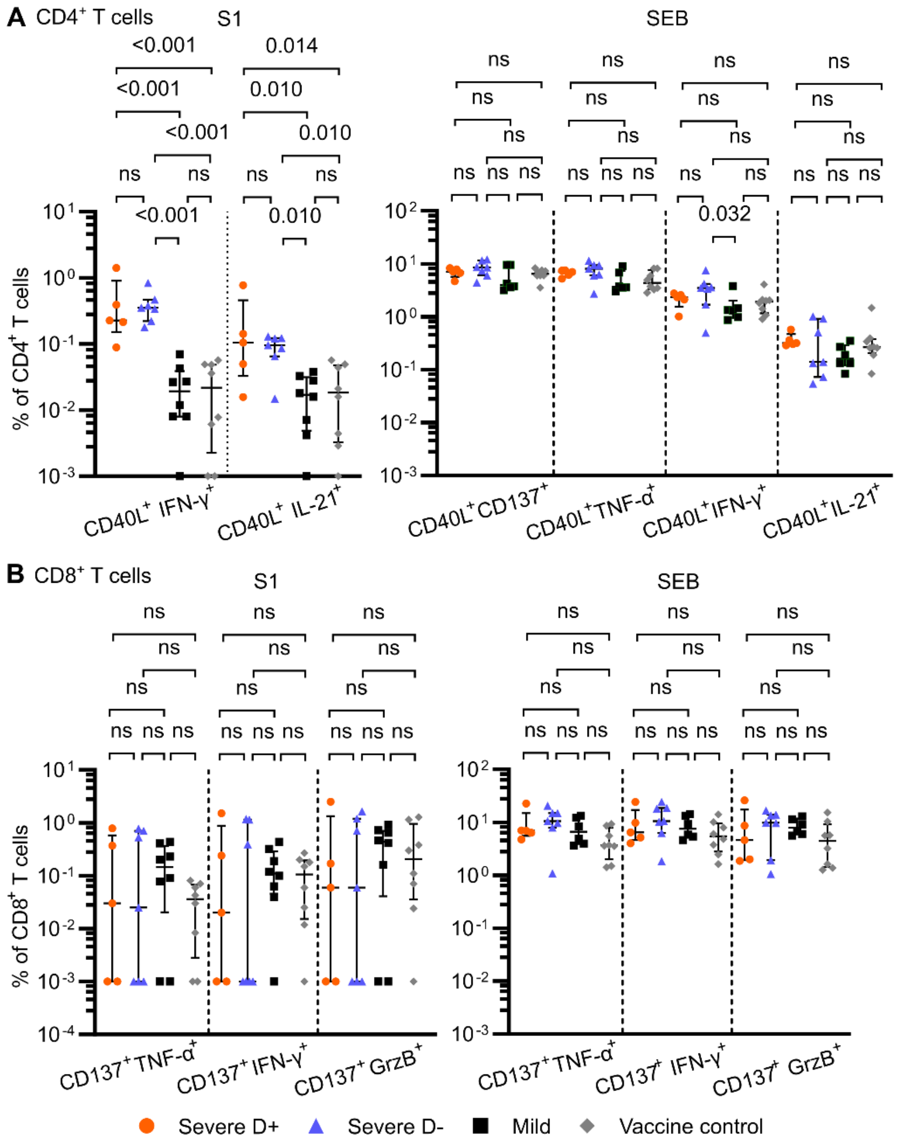

Figure S6: Frequencies of S1- and SEB-reactive CD4⁺ and CD8⁺ T cells after booster immunization. Reactive T-cells were measured in severely affected COVID-19 patients treated with dexamethasone (D⁺, red) or without (D⁻, blue) and mildly affected patients (black) after receiving one dose of a SARS-CoV-2 vaccination. Vaccine controls (gray) without history of COVID-19 who received two doses of a SARS-CoV-2 mRNA vaccination were included as comparison. A) Frequencies of additional marker combinations for S1-reactive CD4+ T cells (CD40L⁺ IFN-γ⁺ and CD40L⁺ IL-21⁺) are shown as percentages of total CD4⁺ T cells (left) and all marker combinations are shown for SEB-reactive CD4⁺ T cells (right). B) Frequencies of S1- (left) and SEB-reactive (right) CD8⁺ T cells (CD137⁺ IFN-γ⁺, CD137⁺ TNF-α⁺ and CD137⁺ GrzB⁺) are shown as percentages of total CD8⁺ T cells. Medians and IQR are shown. Statistical testing was performed using Kruskal-Wallis test with Conover's test for multiple comparisons.

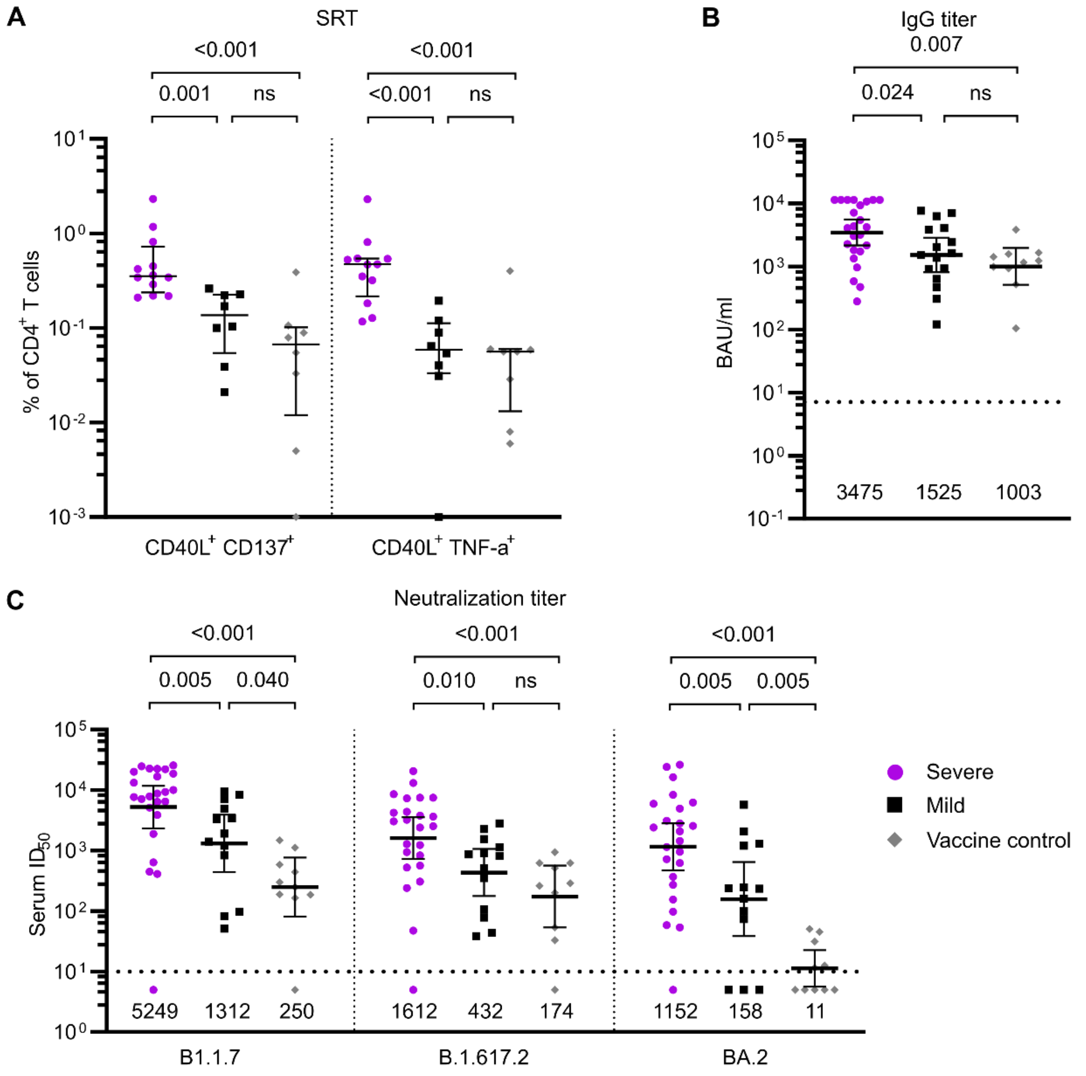

Figure S7: T cell and antibody response after a single immunization is higher in severely affected COVID-19 patients than mildly affected patients and vaccine controls. Comparison of spike reactive T cells (panel A) and antibody response (B,C) in mildly affected patients (black), pooled severely affected patients with and without dexamethasone treatment (purple) who received one dose of a SARS-CoV-2 vaccine and controls without history of COVID-19 who received two doses of mRNA vaccine (gray). A) Frequencies of S1-reactive CD40L$^+$ CD137$^+$ (left) and CD40L$^+$ TNF-α^+ (right) of total CD4$^+$ T cells. Medians and IQR are shown. B) RBD-IgG titers and C) serum ID_{50} titers against B.1.1.7 and B.1.617.2 and BA.2 pseudovirus variants. Geometric means with 95% CI are shown. Black numbers below the dotted lines indicate geometric means, dotted lines indicate cutoff for reactivity/neutralization. Statistical testing for all panels was performed using Kruskal-Wallis-Test with Conover's test for multiple comparisons.

Supplemental Acknowledgements

Members of the Pa-COVID study group :

Victor M Corman[9,10,11], Christian Drosten[9,10], Barbara Mühlemann[9,10], Tatjana Schwarz[9,10], Terry C Jones[9,10], Norbert Suttorp[1], Stefan Hippenstiel[1], Sascha S. Haenel[1], Bettina Temmesfeld-Wollbrück[1], Holger Müller-Redetzky[1], Alexander Uhrig[1], Daniel Grund[1], Christoph Ruwwe-Glösenkamp[1], Miriam S. Stegemann[1], Katrin M. Heim[1], Ralf H. Hübner[1], Bastian Opitz[1], Kai-Uwe Eckardt[12], Martin Möckel[13], Felix Balzer[14], Claudia Spies[14], Steffen Weber-Carstens[14], Frank Tacke[15], Chantip Dang-Heine[6], Michael Hummel[16], Georg Schwanitz[17], Uwe D. Behrens[17], Maria Rönnefarth[6], Sein Schmidt[6], Alexander Krannich[6] and Christof von Kalle[6] (set up and realization of the study platform); Linda Jürgens[1], Malte Kleinschmidt[1], Sophy Denker[6,18], Moritz Pfeiffer[1], Belén Millet Pascual-Leone[1], Luisa Mrziglod[1], Felix Machleidt[1], Sebastian Albus[1], Felix Bremer[1], Jan-Moritz Doehn[1], Tim Andermann[1], Carmen Garcia[1], Philipp Knape[1], Philipp M. Krause[1], Liron Lechtenberg[1], Yaosi Li[1], Panagiotis Pergantis[1], Till Jacobi[1], Teresa Ritter[10], Berna Yedikat[1], Lennart Pfannkuch[1], Ute Kellermann[1], Susanne Fieberg[1], Laure Bosquillon de Jarcy[1,9], Anne Wetzel[1], Christoph Tabeling[1,6], Markus C. Brack[1], Moritz Müller-Plathe[1], Jörg Christian Wildberg[1], Jan M. Kruse[12], Daniel Zickler[12], Andreas Edel[14], Britta Stier[12], Roland Körner[12], Nils B. Müller[12], and Philipp Enghard[12] (obtaining informed consent and biosamples); Nadine Olk[1], Willi M. Koch[1], Alexandra Horn[1], Saskia Zvorc[6], Lucie Kretzler[6], Lil A. Meyer-Arndt[19], Linna Li[6], and Isabelle Wirsching[6] (data collection); Denise Treue[16], Dana Briesemeister[16], Jenny Schlesinger[16], Birgit Sawitzki[20], Kai Pohl[1], Philipp Georg[1], Daniel Wendisch[1], Anna L. Hiller[1] and Sophia Brumhard[1] (contributing to testing and biobanking of samples).

9) Institute of Virology, Charité-Universitätsmedizin Berlin, corporate member of Freie Universität Berlin and Humboldt-Universität zu Berlin, 10117 Berlin, Germany

10) German Centre for Infection Research (DZIF), 10117 Berlin, Germany

11) Labor Berlin-Charité Vivantes GmbH, 13353 Berlin, Germany

12) Institute of Nephrology and Internal Intensive Care Medicine, Charité – Universitätsmedizin Berlin, corporate member of Freie Universität Berlin and Humboldt-Universität zu Berlin, 10117 Berlin, Germany

13) Division of Emergency Medicine and Department of Cardiology, Berlin, Germany, Charité – Universitätsmedizin Berlin, corporate member of Freie Universität Berlin and Humboldt-Universität zu Berlin, 10117 Berlin Germany

14) Department of Anesthesiology and Operative Intensive Care Medicine (CCM, CVK), Berlin, Germany, Charité – Universitätsmedizin Berlin, corporate member of Freie Universität Berlin and Humboldt-Universität zu Berlin, 10117 Berlin, Germany

15) Department of Hepatology and Gastroenterology, Berlin, Germany, Charité – Universitätsmedizin Berlin, corporate member of Freie Universität Berlin and Humboldt-Universität zu Berlin, 10117 Berlin, Germany

16) Central Biobank Charité (ZeBanC), Institute of Pathology, Berlin, Germany Charité – Universitätsmedizin Berlin, corporate member of Freie Universität Berlin and Humboldt-Universität zu Berlin, 13353 Berlin, Germany

17) Clinical Study Center, Berlin, Germany, Charité – Universitätsmedizin Berlin, corporate member of Freie Universität Berlin and Humboldt-Universität zu Berlin, 10117 Berlin, Germany

18) Department of Hematology, Oncology and Tumor Immunology, Berlin, Germany, Charité – Universitätsmedizin Berlin, corporate member of Freie Universität Berlin and Humboldt-Universität zu Berlin, 10117 Berlin, Germany

19) Department of Neurology, Berlin, Germany, Charité – Universitätsmedizin Berlin, corporate member of Freie Universität Berlin and Humboldt-Universität zu Berlin, 10117 Berlin, Germany

20) Institute of Medical Immunology, Charité – Universitätsmedizin Berlin, corporate member of Freie Universität Berlin and Humboldt-Universität Zu Berlin, 10117 Berlin, Germany
